# Tuning interfacial proton transfer for directing oxygen reduction reaction toward hydrogen peroxide

**DOI:** 10.1093/nsr/nwaf390

**Published:** 2025-09-15

**Authors:** Yu Fan, Hao Chen, Wangxin Ge, Xiaodong Zhou, Haiyan Wang, Hongliang Jiang, Chunzhong Li

**Affiliations:** Key Laboratory for Ultrafine Materials of Ministry of Education, School of Chemical Engineering, East China University of Science and Technology, Shanghai 200237, China; Shanghai Engineering Research Center of Hierarchical Nanomaterials, School of Materials Science and Engineering, East China University of Science and Technology, Shanghai 200237, China; Key Laboratory for Ultrafine Materials of Ministry of Education, School of Chemical Engineering, East China University of Science and Technology, Shanghai 200237, China; Shanghai Engineering Research Center of Hierarchical Nanomaterials, School of Materials Science and Engineering, East China University of Science and Technology, Shanghai 200237, China; Key Laboratory for Ultrafine Materials of Ministry of Education, School of Chemical Engineering, East China University of Science and Technology, Shanghai 200237, China; Key Laboratory for Ultrafine Materials of Ministry of Education, School of Chemical Engineering, East China University of Science and Technology, Shanghai 200237, China; Centre of Henan Province for Green Manufacturing of Fine Chemicals, Key Laboratory of Green Chemical Media and Reactions, Ministry of Education, School of Chemistry and Chemical Engineering, Henan Normal University, Xinxiang 453007, China; Key Laboratory for Ultrafine Materials of Ministry of Education, School of Chemical Engineering, East China University of Science and Technology, Shanghai 200237, China; Key Laboratory for Ultrafine Materials of Ministry of Education, School of Chemical Engineering, East China University of Science and Technology, Shanghai 200237, China; Shanghai Engineering Research Center of Hierarchical Nanomaterials, School of Materials Science and Engineering, East China University of Science and Technology, Shanghai 200237, China; Department of Chemical Engineering, School of Chemistry and Chemical Engineering, Shanghai Jiao Tong University, Shanghai 200240, China

**Keywords:** proton transfer, H_2_O_2_ electrosynthesis, solvation structure, hydrogen-bond network, electrolyte

## Abstract

Proton transfer at the electrified interface plays a pivotal role in proton-coupled electron transfer (PCET) reactions. However, tuning the interfacial proton transfer through the electrolyte remains a largely unexplored yet effective approach for boosting electrochemical performance. Here, we demonstrate that the chelation strength of chelating molecules can serve as a criterion for selecting alkaline electrolyte additives to direct the oxygen reduction reaction (ORR) toward hydrogen peroxide (H_2_O_2_) electrosynthesis. We reveal that chelating molecules enter the solvation shell of cations, disrupting the long-range connectivity of hydrogen-bond networks by forming rigid near-range hydrogen bonds. The hydrogen-bond networks serve as a channel for proton transfer through hopping. These reshaped hydrogen-bond networks slow down the proton transfer process. Subsequently, ethylenediaminetetraacetic acid (EDTA), with its high chelation strength, finely regulates proton availability at the electrified interface. This modulation effectively decelerates the 4e^−^-involved PCET kinetics, steering the ORR toward the 2e^−^ pathway of a lower energy barrier. As a result, EDTA-containing electrolytes achieve significantly higher H_2_O_2_ selectivity and Faradaic efficiency than other systems. This work underscores the importance of the interfacial hydrogen-bond networks in electrochemical reaction kinetics and could guide the design of electrolytes for various electrochemical reactions.

## Teaser

This study reports that ethylenediaminetetraacetic acid and its analogs, featuring strong chelation ability with metal cations, are selected as electrolyte additives to reshape interfacial hydrogen-bond network for boosting electrosynthesis of hydrogen peroxide.

## INTRODUCTION

Hydrogen peroxide (H_2_O_2_), a green and benign oxidant, is extensively utilized in bleaching, wastewater treatment, and chemical synthesis, with its global demand experiencing rapid growth [1–4]. The current anthraquinone process for industrial H_2_O_2_ production is energy- and waste-intensive, necessitating a centralized infrastructure. It raises concerns about the safety of the transporting and storing of H_2_O_2_ solutions [5,6].

Electrosynthesis through a 2e^−^ oxygen reduction reaction (ORR) makes it possible to realize on-site production of H_2_O_2_, requiring only sufficient oxygen, water, and renewable electricity [7,8]. However, compared to the 2e^−^ ORR, the competing 4e^−^ ORR to produce water is thermodynamically more favorable, resulting in sluggish kinetics and low selectivity for H_2_O_2_ production [9–11]. Therefore, it is imperative to improve the activity and selectivity of 2e^−^ ORR to achieve sustainable mass production of H_2_O_2_.

As is known that electrochemical reactions are identified to occur in the electric double layer (EDL) at the interface between the catalyst surface and electrolyte solution [12,13], modulation of the EDL microstructure is the key to promote electrochemical performance [14–16]. In the EDL, the hydrogen-bond networks among water molecules facilitate proton transfer through hopping, thus impacting the kinetics of the proton-coupled electron transfer (PCET) process, which in turn determines the performance of electrochemical reactions [17–20]. Chen *et al.* revealed that high connectivity of hydrogen-bond networks increased hydrogen transfer ability, thus being responsible for the rapid kinetics of hydrogen electrocatalysis [19]. By regulating the hydrogen-bond networks, it is feasible to achieve high selectivity for the 2e^−^ ORR while suppressing the excessive 4e^−^ hydrogenation process.

Despite the electrochemical inactivity of cations in the EDL, they significantly affect the structures of interfacial water [21,22]. Some electrolyte additives have been documented to disrupt the initial hydrogen-bond networks by altering the solvation shell of K^+^ in alkaline ORR systems, thereby affecting PCET kinetics and ORR selectivity [17,23,24]. Considering the chelation between chelating agents (CAs) with metal ions, it is postulated that modulating the chelation strength could alter cationic solvation chemistry in alkaline solutions [25,26]. This modulation can reconstruct interfacial hydrogen-bond networks during the electrochemical process, and then affect the activity and selectivity of the ORR [25–28]. Regrettably, the relationship between the nature of CA and ORR performance remains underexplored, leaving the molecular-level understanding unclear.

In this work, we investigated the effect of interfacial hydrogen-bond networks on proton transfer and ORR kinetics of carbon catalysts in KOH solutions. A series of molecules featuring different chelating ability with alkali metal ions were selected as electrolyte additives, such as acetic acid (Ac), glycine (Gly), nitrilotriacetic acid (NTA), and ethylenediaminetetraacetic acid (EDTA). Using density functional theory (DFT) calculations and molecular dynamics (MD) simulations, our study demonstrated that chelating molecules effectively penetrate the solvation shell of K^+^ ions, thereby disrupting their hydrogen-bond networks and impeding proton transfer. Notably, the selectivity for the 2e^−^ ORR is enhanced in the presence of CA with EDTA, and exhibits the highest chelation strength thus yielding the most significant promotion. *In situ* vibrational spectroscopy and electrochemical impedance spectroscopy (EIS) provided mechanistic insights into the adjusted PCET kinetics, which are controlled by mass transfer processes in EDTA-containing electrolytes. Additionally, an insufficient proton supply enhances H_2_O_2_ electrosynthesis. *Ab initio* molecular dynamics (AIMD) simulations further revealed the underlying mechanism governing the ORR process.

## RESULTS AND DISCUSSION

### Solvation structure and hydrogen-bond networks in KOH-H_2_O-CA electrolyte

**Figure 1.**
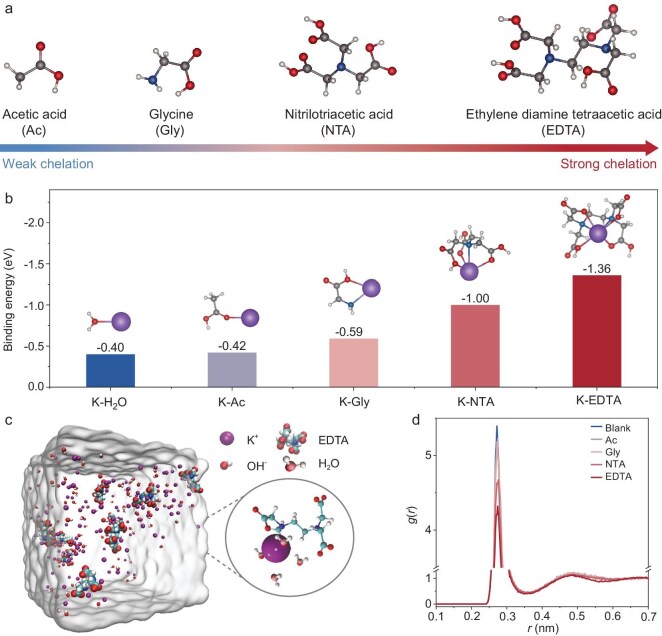
Solvation structure. (a) Chemical formulae of different chelating agents. (b) Binding energies of K-H_2_O and various K-CA, and the corresponding structures. (c) Snapshots of MD simulations for the solvation structure of K^+^ in EDTA-containing electrolytes. (d) The radial distribution functions of K^+^-O_water_ in KOH electrolytes with and without CA collected from MD simulations.

Figure 1a shows the chemical formulae of four molecules whose chelation ability with alkaline-earth metal ions increases in the order of Ac < Gly < NTA < EDTA, which is related to the stability of the generated coordination compound. To investigate the chelation effect on the solvation chemistry of K^+^, DFT calculations were first performed (see Computational methods in Supporting Information). Figure 1b illustrates the binding energy (BE) between K^+^ and CA, which is consistently higher compared to the binding energy between K^+^ and a H_2_O molecule (K-H_2_O BE = −0.40 eV, K-Ac BE = −0.42 eV, K-Gly BE = −0.59 eV, K-NTA BE = −1.00 eV, K-EDTA BE = −1.36 eV). This indicates that K^+^ in the KOH-H_2_O-CA electrolyte preferentially coordinates with CA over H_2_O, signaling a change in the solvation environment of K^+^ compared to that in the KOH-H_2_O electrolyte. Moreover, a chelating molecule with higher chelation ability results in a higher BE value, which suggests a promising method for regulating ion-solvation chemistry by focusing on the chelation ability of CA. To further reveal the theoretical structures of various systems with and without chelating molecules, MD simulations were conducted (Table S1). Snapshots of the KOH-H_2_O and KOH-H_2_O-CA electrolyte are shown in Fig. 1c and Fig. S1. Upon the addition of CA, the electrolyte displays huge aggregation due to the formation of coordination complexes between K^+^, CA, and water molecules. Figure 1d shows the radial distribution function (RDF) of K^+^-O_water_ of various systems. Chelating molecules are found to replace H_2_O molecules in the first solvation shell of K^+^ cations. As the chelating energy of the additive increases, the coordination number between K^+^ and H_2_O in the KOH-H_2_O-Ac electrolyte gradually decreases, especially in the EDTA system (Fig. S2 and Table S2). This suggests that more water molecules are replaced, which is consistent with the BE results of DFT calculations. Comparing the RDFs of K^+^-O_water_ (0.275 nm) and K^+^-O_EDTA_ (0.265 nm) in the EDTA system indicates that EDTA is closer to K^+^ (Fig. S3). These findings suggest that introduction of EDTA can seriously affect the interfacial water structure and its ability to generate a distinct hydrogen-bond environment. This modification impacts the activity of water molecules, which serve as the proton source for H_2_O_2_ [28–31].

**Figure 2.**
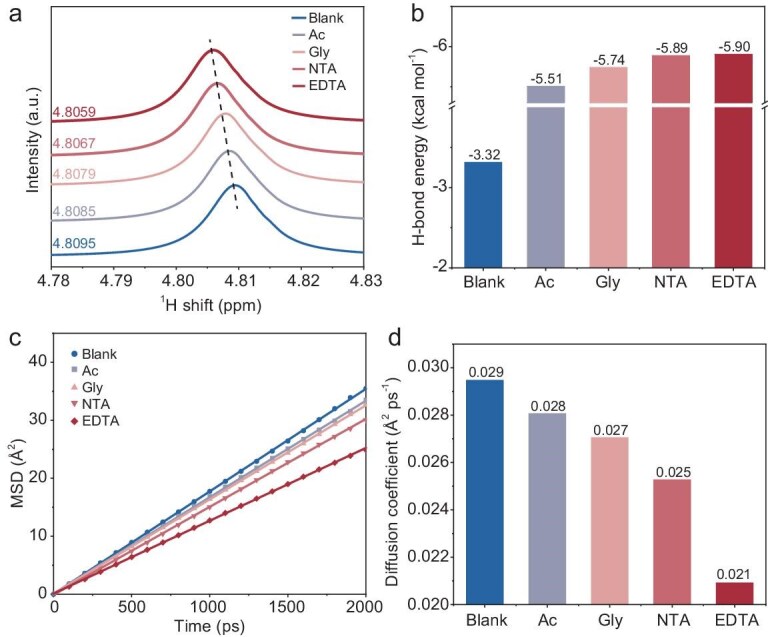
Hydrogen-bond networks. (a) Experimental ^1^H NMR spectra in 0.1 M KOH electrolytes with and without 1 mM CA. (b) Hydrogen-bond energies between H_2_O and CA. (c) Mean square displacements. (d) The corresponding diffusion coefficients of protons in KOH electrolytes with and without CA.

Further analysis was conducted on hydrogen-bond structures within various electrolytes. From the ^1^H nuclear magnetic resonance (NMR) spectra in Fig. 2a, an evident upfield shift of the ^1^H peak is observed with the addition of chelating molecules, indicating increased electron density on hydrogen. This change is associated with CA modifying the solvation sheath, releasing water molecules initially bound to K^+^ [32]. These displaced water molecules can then interact with the chelating molecules, possibly through CA-H_2_O hydrogen bonds. To further support this conclusion, we calculated the strength of CA-H_2_O hydrogen bonds and compared it with the H_2_O-H_2_O interactions in blank alkaline solutions. As shown in Fig. 2b, the CA-H_2_O interactions are remarkably stronger than H_2_O-H_2_O interactions, and the strength of hydrogen bonds increases with increasing chelating ability. We can infer that the addition of CA into the KOH-H_2_O electrolyte creates a high rigidity of short-range CA-H_2_O hydrogen bonds. This disrupts the long-range connectivity of hydrogen-bond networks within H_2_O molecules and thus could hinder proton migration [33,34]. The effect is more significant with higher chelating ability as well as the increase in CA concentration (Fig. S4). To gain deep insight into the proton transfer kinetics in different electrolytes, the mean square displacements (MSDs) were plotted in Fig. 2c and the corresponding diffusion coefficients (Fig. 2d, Table S3) were calculated based on Einstein’s diffusion law (see Computational methods in Supporting Information) [35]. The diffusion coefficients exhibit a gradual decrease with increasing chelation ability of CA, indicating restricted proton mobility over short length scales. This restriction is associated with strengthened hydrogen bonds, which raise the energy barrier for hydrogen bond breakage during proton diffusion [31,36].

The above results underline that chelating molecules successfully enter the solvation shell of K^+^, thereby disrupting the long-range connectivity of hydrogen-bond networks by forming rigid short-range CA-H_2_O hydrogen bonds and reducing proton mobility. This modification will undoubtedly affect the water-involved PCET process in ORRs. It motivates us to regulate the hydrogenation of the ORR through solvation chemistry in order to boost H_2_O_2_ production.

### CA effect on electrochemical ORR performance

**Figure 3.**
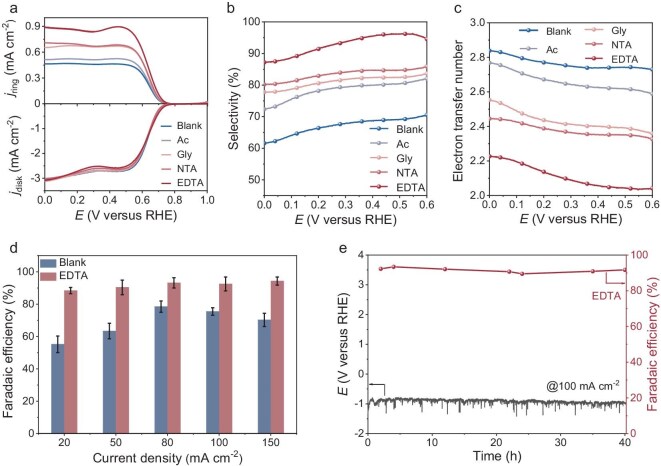
ORR performance. (a) LSV curves of CB catalysts at a scan rate of 5 mV s^−1^ in 0.1 M KOH with and without 4 mM CA. The risk current density *j*_ring_ represented the H_2_O_2_ oxidation current. (b) H_2_O_2_ selectivity of CB catalysts. (c) Corresponding electron transfer number. (d) H_2_O_2_ electrosynthesis performance in a flow cell in 0.1 M KOH electrolytes with and without 4.0 mM EDTA at different current densities. (e) Stability of GDE in the 0.1 M KOH electrolyte with 4.0 mM EDTA at 100 mA cm^−2^.

The role of different KOH-H_2_O-CA electrolytes in modulating the ORR was probed using commercial carbon black (CB), a typical ORR catalyst in alkaline electrolytes, as the model catalyst (Fig. S5). The ORR activity and H_2_O_2_ selectivity of CB with and without CA were evaluated on a rotating ring-disk electrode (RRDE) in O_2_-saturated 0.1 M KOH electrolytes (see Experimental procedures in Supporting Information). The ring electrode was maintained at 1.2 V versus a reversible hydrogen electrode (RHE) to monitor H_2_O_2_ selectivity throughout the test process. As shown in Fig. 3a, the electrolytes with CA addition display superior ORR activity toward H_2_O_2_ compared to the blank KOH-H_2_O electrolyte, as indicated by the increased H_2_O_2_ oxidation current (j_ring_). Figure 3b and c illustrate the calculated H_2_O_2_ selectivity and electron transfer number (n), demonstrating significant variations in 2e⁻ ORR activity among different CA additives. Notably, EDTA additives in a KOH-H_2_O electrolyte maximizes H_2_O_2_ selectivity (exceeding 80%), achieving an n value closest to 2, followed by NTA, Gly, and Ac, respectively. Meanwhile, the addition of EDTA does not change the double-layer capacitance, that is, the electrochemically active surface area does not change [37], which could substantiate the discrepancy of EDL microstructure at blank KOH-H_2_O and EDTA-mediated ORR interfaces (Fig. S6) [23]. These results corroborate with previous predictions, verifying that strong EDTA-H_2_O interactions disrupt the long-range connectivity of hydrogen-bond networks. Consequently, this disruption leads to an inadequate proton supply environment, which is insufficient for excessive hydrogenation [24]. Moreover, the effect of EDTA concentration on H_2_O_2_ selectivity was investigated (Fig. S7). We observed that the selectivity for H_2_O_2_ increased with increasing EDTA concentration in the range of 0 to 4 mM. However, excessive addition of EDTA (5 mM) is detrimental to H_2_O_2_ production and causes a significant negative shift in the onset potential. This shift is attributed to the inhibition of proton transfer, underscoring the importance of controlled connectivity within hydrogen-bond networks [38,39]. This phenomenon is further corroborated by our studies with diphenylenetriamine pentaacetic acid (DTPA), where its stronger chelating capacity [40] leads to even more pronounced suppression effects—not only exacerbating the onset potential shift but also reducing overall catalytic current (Fig. S8). This reveals a critical balance: although proper chelator levels improve selectivity, excessive chelation strength (as with DTPA) or concentration (5 mM EDTA) disrupts proton transfer in H_2_O_2_ production. Furthermore, the enhancement in H_2_O_2_ selectivity induced by EDTA was also observed in NaOH electrolytes (Fig. S9), indicating that this strategy is applicable to monovalent cation systems beyond K^+^. This consistency underscores the universal role of chelators in modulating the interfacial microenvironment and steering the ORR pathway toward selective H_2_O_2_ production in alkaline media. However, this chelation-based approach exhibits significant environmental specificity. The enhancement of H_2_O_2_ selectivity by EDTA is highly dependent on alkaline environments, and it shows no effect under acidic or neutral conditions (Figs S10 and S11). This is primarily due to the protonation of EDTA’s carboxyl groups at low pH, which weakens its chelating ability, and a shift in proton transfer mechanism from water-mediated to hydronium-dominated. In seawater containing Ca^2+^/Mg^2+^, EDTA is readily sequestered by these competing ions and may form precipitates that block active sites. This strategy is only effective in alkaline electrolytes where interfacial water molecules and cation solvation structures play a dominant role in the reaction system. The use of other carbon-based catalysts, such as acetylene black and Ketjen black, has similarly demonstrated performance enhancement with EDTA addition, thereby illustrating the general applicability of EDTA-modified electrolytes in enhancing H_2_O_2_ selectivity (Figs S12–S15). In contrast, on metal-based catalysts like Pt/C, EDTA adsorbs onto active sites without altering the inherent 4e^−^ pathway preference (Figs S16 and S17), highlighting the catalyst-specific behavior of electrolyte additives.

To explore the possibility for practical use, we performed electrolysis in a three-electrode flow cell with a gas diffusion electrode (GDE) for H_2_O_2_ generation. The quantification of H_2_O_2_ was determined utilizing a colorimetric quantification method (Fig. S18). Figure 3d shows the H_2_O_2_ Faradaic efficiency of GDE in the KOH electrolytes with and without EDTA. From 20 to 150 mA cm^−2^, the Faradaic efficiency for H_2_O_2_ generation is between 88.4% and 94.3% in KOH-H_2_O-EDTA electrolytes, which is much higher than that in KOH-H_2_O electrolytes. Moreover, the H_2_O_2_ Faradaic efficiency can maintain ∼90% for a 40-h duration test at 100 mA cm^−2^, indicating a stable and efficient H_2_O_2_ electrosynthesis process in KOH-H_2_O-EDTA electrolytes (Fig. 3e). The electrochemical stability of the CB catalyst in the EDTA-containing electrolyte was evaluated using the RRDE device. As shown in Fig. S19, the system demonstrated stable current densities and maintained high H_2_O_2_ selectivity during 18 h of continuous operation. Furthermore, analysis of the electrolyte before and after the reaction by NMR confirmed that EDTA did not undergo decomposition into other products and maintained its structural integrity throughout the entire testing period (Fig. S20). Overall, based on mechanism analysis and experimental verification, EDTA addition has a significant catalytic enhancement effect on the electro-synthesis efficiency of H_2_O_2_.

### PCET kinetics of ORR in KOH-H_2_O-EDTA electrolyte

**Figure 4.**
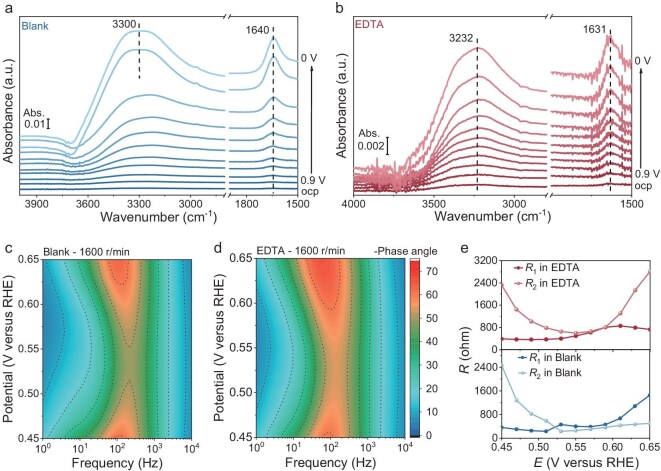
PCET kinetics for ORR process. (a) *In situ* ATR-SEIRAS spectra of the KOH electrolyte (Blank). (b) *In situ* ATR-SEIRAS spectra of the KOH electrolyte containing 4 mM EDTA (EDTA). (c) Bode plots of 0.1 M KOH electrolytes with a working electrode rotation at a rate of 1600 r/min. (d) Bode plots of 0.1 M KOH electrolytes containing 4 mM EDTA at 0.65–0.45 V_RHE_ with a working electrode rotation rate of 1600 r/min. (e) Fitting resistance from EIS measurements in 0.1 M KOH and 0.1 M KOH with 4 mM EDTA, recorded at a working electrode rotation rate of 1600 r/min.

According to current theory, the ORR mechanism involves PCET steps, and the PCET kinetics can be regulated by altering the hydrogen-bond structures in the EDL [9,19,41]. *In situ* attenuated total reflection surface-enhanced infrared absorption spectroscopy (ATR-SEIRAS) is well suited to probing interfacial water for investigation of the hydrogen-bond environment (Fig. S21). Figure 4a and b shows the *in situ* ATR-SEIRAS spectra of the KOH electrolytes with and without EDTA, respectively. As the bias potential increases, the O─H stretching vibration (ν-OH, 2800–3800 cm^−1^) and the H─O─H bending vibration (δ-HOH, ∼1630 cm^−1^) of water gradually enhances, revealing water enrichment at the electrified interface [42]. An obvious redshift from ∼3300 to ∼3232 cm^−1^ of the ν-OH band can be observed for the KOH electrolytes with EDTA compared to blank electrolytes. Previous studies reveal that the ν-OH peak of water can be deconvoluted into three distinct components: strong hydrogen-bond water (3200 cm^−1^), weak hydrogen-bond water (3400 cm^−1^), and free water (3600 cm^−1^), according to three O─H stretching modes of interfacial water [43]. It can be found from the fitting results (Fig. S22) that the interfacial water is mainly composed of strong and weak hydrogen-bond water. Apparently, the addition of EDTA increases the percentage of strong hydrogen-bond water molecules in the EDL (Tables S4 and S5). Therefore, the redshift of the ν-OH band after EDTA addition evidences strong EDTA-H_2_O interactions. The lower band intensity observed in KOH-H_2_O-EDTA electrolytes compared to blank KOH-H_2_O electrolytes can be attributed to a reduction in water molecules. This finding aligns with theoretical calculations indicating that strong K-EDTA and EDTA-H_2_O interactions not only decrease the number of water molecules in the solvation shell of K^+^ but also disrupt the connectivity of hydrogen-bond networks among water molecules, thus reducing interfacial water content. Consequently, the introduction of EDTA slows down proton transport kinetics in the EDL, thereby inhibiting excessive hydrogenation and the subsequent 4e^−^ ORR process [9,24,28].

To further elucidate the interfacial charge transfer resistance and electrochemical kinetics during the ORR process, EIS measurements were conducted at different potentials. To avoid interference from diffusion effects in porous electrode materials, we used a bare glassy carbon electrode for the EIS test (Fig. S23). Figure 4c, d and Figs S24, S25 show the Bode plots of 0.1 M KOH electrolytes with and without EDTA under different electrode rotation rates. The increase of rotation rates from 900 to 1600 r/min results in an obvious decrease of phase angle (Ø_peak_) in low frequency, which is associated with accelerated mass transfer [44,45]. Compared to the blank system, the EDTA-containing system displays higher Ø_peak_ and thus relatively slower kinetics throughout the potential change. Moreover, the Ø_peak_ decreases first with the increase of the bias potential, reaches the minimum value ∼0.55 V_RHE_, and then gradually increases (Fig. 4c, d and Fig. S26). This variation is consistent with previous research involving competition between electron transfer resistance (R_1_) and mass transfer resistance (R_2_) [9]. By fitting the equivalent circuit diagram (Fig. S27), we conducted a closer analysis of the R_1_ and R_2_ contributions to the overall Faradaic impedance (R) to better understand the different kinetic properties. For the blank KOH electrolyte in the range of 0.65 to 0.45 V_RHE_, the ORR is controlled first by the electron transfer process and then by mass transfer process due to higher R_1_ initially and R_2_ later (Fig. 4e). For the EDTA-modified KOH electrolytes, since R_2_ is higher than R_1_ almost throughout the potential range, the ORR is always controlled by the mass transfer process (Figs S28 and S29). In the meantime, EDTA addition results in a significant increase in R_2_ and little difference in R_1_. It suggests that EDTA addition slows down the PCET kinetics by primarily impeding the proton transport process and having minimal influence on the electron transfer process. This effect reduces proton availability, resulting in sluggish 4e^−^-involved PCET kinetics, and drives the ORR toward a 2e^−^ pathway with less proton participation, thereby achieving efficient 2e^−^ ORR activity. To distinguish between the contributions of proton transport limitation and the intrinsic activity of the catalyst, we performed kinetic isotope effect (KIE) experiments comparing reaction rates in H_2_O- and D_2_O-based electrolytes (see details in Experimental procedures). The 0.1 M KOH-H_2_O electrolyte containing 4 mM EDTA exhibits a notably enhanced KIE, particularly at potentials exceeding 0.5 V_RHE_, demonstrating that proton transfer kinetics becomes critical to the rate-determining step (Fig. S30). This voltage-dependent divergence in KIE behavior conclusively confirms EDTA’s role in restructuring the reaction pathway by introducing proton-transfer-dependent bottlenecks.

### Theoretical insights into interfacial electrochemical process

**Figure 5.**
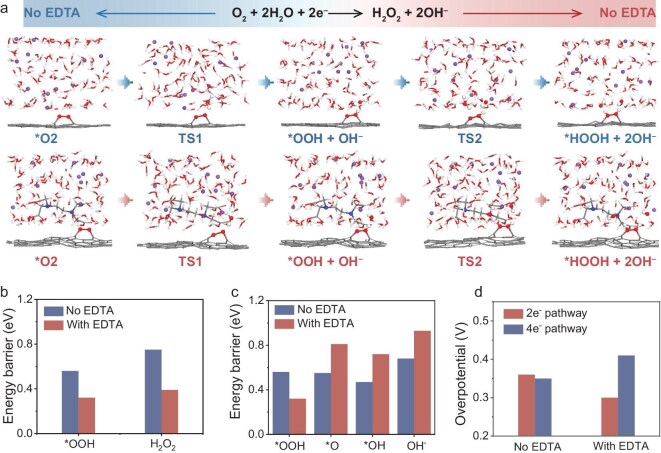
Theoretical insights into electrochemical processes at KOH-H_2_O and KOH-H_2_O-EDTA interfaces. (a) Representative snapshots of 2e^−^ ORR pathway at KOH-H_2_O and KOH-H_2_O-EDTA interfaces (TS: transition state). The C, O, H, N and K are colored in grey, red, white, blue and purple, respectively. Energy barrier of 2e^−^ (b) and 4e^−^ (c) ORR at KOH-H_2_O and KOH-H_2_O-EDTA interfaces. (d) Simulated overpotential values for the 2e⁻ and 4e⁻ ORR pathways at the KOH-H₂O and KOH-H₂O-EDTA interfaces.

*Ab initio* molecular dynamics (AIMD) simulations were further performed to probe the origin of the enhancement of ORR performance. We simulated the 2e^−^ ORR pathway in both EDTA-containing and EDTA-free KOH electrolytes, and the corresponding structures are illustrated in Fig. 5a. Starting with O_2_ adsorption (*O_2_) on the carbon catalyst surface, the *O_2_ intermediates obtain protons from the interfacial water molecules to transform into *OOH intermediates, and continue to obtain protons to generate H_2_O_2_. It is noted that each reaction intermediate along the 2e^−^ ORR pathway is much closer to the interfacial water environment in the EDTA-containing system than in the EDTA-free system, and accordingly affects proton supply [46]. Moreover, the statistical distribution of hydrogen bonds along the normal surface direction is plotted in Fig. S31. In the KOH-H_2_O-EDTA system, the hydrogen bonds consist of H_2_O-H_2_O bonds and EDTA-H_2_O bonds. Over a large spatial range from 2.0 to 10.0 Å, the number of H_2_O-H_2_O bonds as well as the total number of hydrogen bonds in the KOH-H_2_O-EDTA system is significantly lower than that in the KOH-H_2_O system. This reduction necessitates higher energy for reconstructing hydrogen-bond networks, thereby significantly impacting the proton transfer kinetics governed by hydrogen-bond jump exchanges [47]. Based on AIMD simulations of the 2e^−^ ORR process, the energy barrier of each step in two systems was calculated (Fig. 5b), which is important in order to appraise the intrinsic catalytic performance. As can be seen, the KOH-H_2_O-EDTA system exhibits much lower energy barriers than the EDTA-free system in both steps along the 2e^−^ process. Meanwhile, the 4e^−^ ORR pathway of EDTA-containing and EDTA-free systems were also considered. The corresponding structures are shown in Figs S32 and S33 and the energy barriers of each step are presented in Fig. 5c. It is evident that the energy barrier for *OOH decomposing to the *O step (0.81 eV) is much higher than that for *OOH obtaining protons to form the H_2_O_2_ step (0.39 eV). This indicates that EDTA facilitates a more facile reaction pathway for 2e^−^ ORR rather than the 4e^−^ process. Figure S34 provides the free energy diagrams of ORR processes at KOH-H_2_O and KOH-H_2_O-EDTA interfaces along 2e^−^ and 4e^−^ pathways. The minimum energy change in free energy diagrams can also be identified as the reaction energy barrier of the respective model compound [48]. In the KOH-EDTA-H_2_O system, the bond lengths of d(O-H) and d(O-OH) shortened, and the bond angle of O–OH increased. These changes indicate that K^+^-EDTA complexation significantly alters the configuration of key intermediates, thereby influencing the reaction pathway, as shown in Fig. S35 [49]. From the diagrams of both systems, the potential determining steps (PDSs) of the 2e^−^ and 4e^−^ process are assigned to the *OOH→*HOOH and *OH release step, respectively. A key distinction lies in the differing PDS energy barrier between EDTA-containing and EDTA-free systems. Specifically, the *OOH→*HOOH step demands a lower energy barrier in the EDTA-containing system, while the *OH release step requires a lower energy barrier in the EDTA-free system. In Fig. 5d, we further compare the overpotentials for KOH-H_2_O and KOH-H_2_O-EDTA systems using a free energy diagram. It shows that the introduction of EDTA can reduce the 2e^−^ ORR overpotential to 0.30 V, while 4e^−^ ORR shows a relatively high overpotential of 0.41 V. These results again reinforce that EDTA modification lowers the PDS energy barrier and elevates 2e^−^ ORR selectivity.

## CONCLUSION

In this work, we have proposed to utilize CA molecules to reshape interfacial hydrogen-bond networks, thereby enhancing the selectivity of 2e^−^ ORR toward H_2_O_2_ in alkaline electrolytes. The DFT calculations and MD simulations show that CA with varying chelating ability can modulate the solvation shell of cations, form rigid short-range hydrogen bonds, and disrupt the long-range connectivity of hydrogen-bond networks among water molecules, thus impeding proton transfer, and the chelation strength greatly impacts this regulation. As a result, EDTA-containing KOH electrolytes achieve much higher H_2_O_2_ selectivity and H_2_O_2_ Faradaic efficiency compared to other CA-containing systems as well as blank KOH electrolytes. EIS and *in situ* ATR-SEIRAS analyses reveal that such interfacial hydrogen-bond networks in EDTA-containing KOH electrolytes significantly affect the mass transfer process, which dominates PCET kinetics to achieve a preferential 2e^−^ ORR pathway. AIMD simulations further elucidate that the EDTA-containing system manifests a significantly diminished energy barrier and reduced overpotential along the 2e^−^ ORR pathway compared to the less favorable 4e^−^ process. These findings established here propose future directions that electrolyte engineering can effectively steer the performance of various electrocatalytic reactions.

## Supplementary Material

nwaf390_Supplemental_File
