# Supplementary material for: Tuning interfacial proton transfer for directing oxygen reduction reaction toward hydrogen peroxide
Source: Natl Sci Rev. 2025 Sep 15;12(11):nwaf390. doi: 10.1093/nsr/nwaf390 (PMC12604015; doi:10.1093/nsr/nwaf390)
Supplement: nwaf390_Supplemental_File [file nwaf390_supplemental_file.pdf]

Supporting Information for

Tuning Interfacial Proton Transfer for Directing Oxygen Reduction Reaction toward Hydrogen Peroxide

Yu Fan, Hao Chen, Wangxin Ge, Xiaodong Zhou, Haiyan Wang, Hongliang Jiang, and Chunzhong Li

Correspondence to: wanghaiyan@htu.edu.cn (Haiyan Wang); jhlworld@ecust.edu.cn (Hongliang Jiang); czli@ecust.edu.cn and czlilab@sjtu.edu.cn (Chunzhong Li)

This PDF file includes:

Experimental procedures

Computational methods

Figures S1 to S35

Tables S1 to S5

1. Experimental procedures

1.1 Chemicals

Carbon Black (CB) was supplied by Macklin (cabot vulcan XC-72R). Potassium hydroxide (KOH, 99.99%) and sodium hydroxide (NaOH, 99.99%) were purchased from Macklin. Acetylene black and Ketjen black were purchased from Aladdin. The chelating agents employed in this study, including acetic acid (Ac, 99.5%), glycine (Gly, 99.5%), nitrilotriacetic acid (NTA, 99.5%), ethylenediaminetetraacetic acid (EDTA, 99.5%), and diethylenetriamine pentaacetic acid (DTPA, 99.5%), were all supplied by Aladdin. The cerous sulfate standard solution ($Ce(SO_4)_2$, 0.1 mol L^{-1}) was obtained from Bolinda. The standard sulphuric acid solution (H_2SO_4, 98 wt.%) was provided by Aladdin. Sodium sulfate ($NaSO_4$, 99.5%) was purchased from Aladdin. The commercial Pt/C (20 wt.%) was obtained from Sinero. Deuterium oxide (D_2O, 99.9%), containing tetramethylsilane (TMS, 0.05 wt.%). was purchased from Energy Chemical. The water mentioned in this work was deionized water (18.2 MΩ cm).

1.2 Electrolyte characterization

The morphology of CB was examined using transmission electron microscopy (TEM, TECNAI G2 F20) and scanning electron microscopy (SEM, Hitachi S-4800). The Raman spectra were acquired using a LabRAM HR Evolution Raman spectrometer (HORIBA Scientific) with a 532 nm laser. Attenuated Total Reflection Surface Enhanced Infrared Absorption (ATR-SEIRA) measurements were performed via a Perkin Elmer Spectrum 100 FT-IR spectrometer. Liquid-state Nuclear Magnetic Resonance spectroscopy were recorded on a Ascend 600 MHz NMR spectrometer (Bruker, Germany). Ultraviolet-visible (UV-Vis) spectra were obtained using a Shimadzu UV-2600 spectrophotometer.

1.3 Electrochemical measurements

Electrochemical measurements were conducted using a three-electrode system controlled by an electrochemical workstation (CHI760E, CH Instruments). A catalyst-loaded rotating ring-disk electrode (RRDE, AFE7R9GCPT, Pine Research Instrumentation) was used as the working electrode. The RRDE comprises a glassy carbon disk electrode with an area of 0.2475 cm^2 and a platinum ring electrode with an area of 0.1866 cm^2. A graphite rod and an Ag/AgCl electrode (RREF0024, Pine Research Instrumentation) were used as the counter and reference electrodes, respectively. To prepare the catalyst ink, 5.0 mg of electrocatalyst was dispersed in a

mixture containing 490 μL of ethanol, 20 μL of Nafion solution (5.0 wt%, Macklin) and 490 μL of deionized water with ultrasonication for 30 minutes. Subsequently, 5.0 μL of the resulting ink was drop casted onto the disk electrode. The electrochemical experiments were performed in O_2-saturated 0.1 M KOH electrolyte. The trace amount of chelating agent (4 mM) was completely dissolved in the KOH solution, and its addition caused a negligible effect on the pH value of KOH solution. Cyclic voltammetry tests were first performed from 0 to 1 VRHE (potentials referenced to the reversible hydrogen electrode, RHE) for 10 cycles at a scan rate of 50 mV s^{-1}. Linear sweep voltammetry (LSV) curves were recorded at 1600 rpm with a scan rate of 5 mV s^{-1}, while the potential of the ring electrode was held at 1.2 V_{RHE}. The potential was calibrated to the RHE reference scale employing the following formulas:

$$E_{RHE} = E_{Ag/AgCl} + 0.0591 \times pH + 0.199 \quad (1)$$

Equations (2) and (3) were used to calculate the electron transfer number (n) and H_2O_2 selectivity (%), where Iring is the ring current, I_{disk} is the disk current, and N_c (0.371) is the collection efficiency of RRDE.

$$H_2O_2 \text{ Selectivity } (\%) = 200 \times \frac{\frac{I_{ring}}{N_c}}{|I_{disk}| + \frac{I_{ring}}{N_c}} \quad (2)$$

$$n = 4 \times \frac{|I_{disk}|}{|I_{disk}| + \frac{I_{ring}}{N_c}} \quad (3)$$

1.4 H_2O_2 concentration measurement

Electrosynthesis of H_2O_2 was conducted using a flow-cell device. This setup comprised an anolyte chamber and a catholyte chamber, each with a window area of 1 cm^2, separated by a Nafion membrane. (Nafion 117, Fuel Cell Store). A gas diffusion electrode (SIGRACET 30T) was used as working electrode. An iridium-plated titanium wire and a Hg/HgO electrode were used as counter and reference electrode, respectively. The conversion formula of Hg/HgO electrode was shown in equations (4).

$$E_{RHE} = E_{Hg/HgO} + 0.0591 \times pH + 0.098 \quad (4)$$

In the flow cell test, a 0.1 M KOH electrolyte solution (100 mL) was circulated through the electrochemical cell utilizing a peristaltic pump with the flow rate maintained at 15 mL min^{-1}. The mass flow controller maintained a constant O_2 flow rate of 30 mL·min^{-1}. The H_2O_2 concentration was measured by determining $Ce(SO_4)_2$

concentration, utilizing a redox mechanism where Ce^{4+} in its yellow form is reduced to colorless Ce^{3+} by H_2O_2[1,2]. UV-Vis spectroscopy was employed to quantify the Ce^{4+} concentrations before and after the reaction at a wavelength of 317 nm. The H_2O_2 concentrations were determined based on the linear relationship between signal intensity and Ce^{4+} concentration (ranging from 0.1 to 0.5 mM). The Faradaic efficiency for H_2O_2 generation was calculated by equation (5).

$$\text{Faradaic efficiency (\%)} = 100 \times \frac{M_{H_2O_2} \times V_{reaction} \times 2 \times F}{Q} \quad (5)$$

where F indicates the Faradaic constant (96485 C mol^{-1}), $M_{H_2O_2}$ represents the H_2O_2 concentrations (mol L^{-1}), Vreation is electrolyte volume (L), and Q is the total charge (C).

1.5 In situ ATR-SEIRAS experiments

The electrochemical experiment was conducted using a customized three-electrode electrochemical single cell. A Pt wire and a saturated Ag/AgCl electrode were used as the counter and reference electrodes, respectively. For the working electrode preparation, CB was loaded onto a chemically deposited polycrystalline Au nanofilm ATR-IR prism through a multi-step process. First, the Si prism surface was polished with 0.05 μm alumina slurry until it achieved hydrophobicity, followed by sequential sonication in water, acetone, and water baths to remove residual particles. The crystal surface was then immersed in 40% NH_4F solution for 5 minutes to eliminate native oxides and create a hydride-terminated surface. Subsequently, the reflecting surface was immersed in a plating solution composed of a mixture of 3 mL 5 wt.% HF and a gold plating solution consisting of $NaAuCl_4 \cdot 2H_2O$ (0.009 M), NH_4Cl (0.03 M), Na_2SO_3 (0.09 M), $Na_2S_2O_3 \cdot 5H_2O$ (0.03 M) and NaOH (0.03 M) at 55 ℃ for 5 minutes to deposit the Au film. Finally, the CB catalyst ink (~0.1 mg cm^{-2}) was coated onto the chemically formed Au nanofilm to complete the working electrode assembly, maintaining electrode preparation standards consistent with those described in the three-electrode system validations. The in situ ATR-SEIRA spectra were recorded by varying the potential from 0.95 to 0.05 V_{RHE}, with an open circuit potential (OCP) of ~1.1 V_{RHE}. The spectral resolution was set at 4 cm^{-1}. Before infrared spectrum acquisition, we conducted a quick CV scan in O_2-saturated electrolyte to remove adsorbed species from the electrode surface. The background spectrum was taken at the OCP, and the experimental infrared spectrum represented the change relative to this background spectrum in the same electrolyte. All spectra were calibrated using OMINIC software, which applied automatic baseline correction and smoothing. By

Gaussian function curve fitting, the water peaks (2800-3800 cm^{-1}) were deconvoluted into three sub-peaks, with the peak positions being predetermined at 3200, 3400, and 3600 cm^{-1}. The intensity of interfacial water molecules was determined by the peak area, while the proportion of various water molecule types was obtained by normalizing this peak area.

1.6 EIS measurement and fitting

The EIS measurements were also performed on the RRDE over a frequency range of 0.1 to 10000 Hz with an applied AC voltage amplitude of 5 mV. The electrolyte in each system should be updated before EIS test to prevent interference from H_2O_2 accumulation during the test. The obtained EIS data were analyzed using ZView2 software. Here, R_s represents the solution resistance, R_1 or R_2 denotes the interface resistance of the ORR. CPE stands for the Constant Phase Element.

1.7 Kinetic isotope effect (KIE) experiments

KIE experiments were performed using 0.1 M KOH and 0.1 M KOD solutions prepared with deionized water (18.2 MΩ cm) and D_2O, respectively[4]. The catalyst coated electrodes were soaked in the electrolyte solution for 30 min before electrochemical experiments. All electrochemical experiments followed the same procedure described above. The kinetic current density (j_k) was calculated using the Koutecky-Levich formula:

$$\frac{1}{j} = \frac{1}{j_k} + \frac{1}{j_L} \quad (6)$$

where j indicates the measured current and j_L is the limiting current. The limiting current was obtained from the Levich equations with the total electron transfer number (n) from the RRDE setup, as directly determining the limiting current for carbonaceous catalysts presents challenges:

$$j_L = 0.62\ n\ FAD_0^{2/3}\omega^{1/2}v^{-1}C_0 \quad (7)$$

where F, A, D_0, ω, υ and C_0 are the Faraday constant (96,485 C mol^{-1}), geometric area of the disk electrode (0.2475 cm^2), diffusion coefficient of O_2 in the electrolyte at 298 K (1.85×10^{-5} cm^2 s^{-1}), electrode rotation rate (1600 rpm), kinematic viscosity (0.89×10^{-2} cm^2 s^{-1}), and O_2 concentration (1.21×10^{-6} mol cm^{-3}).

$$\mathrm{KIE} = k_f^H / k_f^D = \frac{j_k^H n^D F C_{o_2}^{*,D}}{j_k^D n^{DH} F C_{o_2}^{*,H}} \quad (8)$$

Where $\frac{C_{o_2}^{*,D}}{C_{o_2}^{*,H}} = 1.101$ at (298 K), accounting for the difference in the solubility of O_2 in

D_2O and H_2O. Assuming equivalent electron transfer numbers ($n^D = n^H$), the KIE can then be calculated as:

$$KIE=1.101\frac{j_k^H}{j_k^D} \quad (9)$$

2. Computational methods

The density functional theory (DFT) calculations were performed to investigate the surface structure by using the Vienna ab initio simulation package (VASP) code[5]. The projector augmented wave (PAW) method is used to describe the ionic potential and the Perdew-Burke-Ernzerhof (PBE) functional is used to describe the exchange correlation interactions[6,7]. The plane-wave kinetic energy cutoff is 500 eV. A 3×3×3 Monkhorst-Pack k-point mesh is used for the Brillouin zone sampling in all the calculations[8]. The energy convergence criteria for electronic relaxation is 1×10^{-5} eV, and the ionic relaxation is performed until all forces are smaller than 0.01 eV/Å. The free energy(ΔG) of each reduction step was obtained at zero bias potential using

$$\Delta G = \Delta E + \Delta E_{ZPE} - T\Delta S \quad (10)$$

where ΔE was the reaction energy, ΔE_{ZPE} was the difference in zero-point energies, T was the temperature and ΔS is the reaction entropy. The $2e^-$ ORR overpotential is defined as

$$\eta=0.7\ V - \min(4.92 - \Delta G_{OOH}, \Delta G_{OOH} - \Delta G_{HOOH})/e \quad (11)$$

and for $4e^-$ ORR overpotential

$$\eta'=1.23\ V - \min(4.92 - \Delta G_{OOH}, \Delta G_{OOH} - \Delta G_{O}, \Delta G_{O} - \Delta G_{OH}, \Delta G_{OH})/e \quad (12)$$

The ab initio molecular dynamics (AIMD) simulations were performed to elucidate the nature of interfacial environment[9]. The carbon model catalyst was built by a graphene substrate composed of a 6 × 7 supercell. To investigate the influence of EDTA, two pre-equilibrated solvent configurations (157 H_2O molecules, 157 H_2O + 1 EDTA molecules) were inserted on top of the graphene surface respectively and underwent AIMD for 200 ps to equilibrate the two systems. Then, we inserted a K^+ ion in a cavity of the first solvation layer close to the surface, subsequently removing a hydrogen from a water molecule in the outermost water layer and a hydrogen from the EDTA molecule to keep the charge balance, respectively. We carried out AIMD simulations for a total of 200.0 ps with a 1 fs timestep in a canonical NVT (particle number, volume and temperature) ensemble at 300 K regulated by a Nosé–Hoover thermostat. And the total 200 ps of both systems was used to analyze influence of EDTA on the H-bond network. Based on the well-equilibrated interface, the hydrogen-bond numbers were counted along the surface normal direction, and the

reaction barriers for each step were estimated by thermodynamic integration based on Blue Moon ensemble method.

The molecular dynamic (MD) simulations were carried out in the GROMACS simulation package to investigate the microstructures of electrolytes. All the geometry optimizations and energy calculations of each individual compound in MD simulations were performed in the Gaussian 09 software package[10,11]. The B3LYP/6-311G* with GD3BJ dispersion correction was applied for the geometry optimization, and M062X with def2TZVP basis set was used for energy and frequency calculation[12,13]. The MD solvation model was applied in all the calculations. The 1 M KOH aqueous electrolyte systems were simulated to investigate the effect of different anion on the solvation shell. The CHARMM36 all atomic force field was used, including the original force field parameters for all the elements. The topological files and force field of five anions were generated by CGenFF. The simulations were performed in the NPT ensemble with constant pressure (1 atm) and temperature (300 K), and ran for 10 ns with the time step of 1 fs. The temperature and pressure coupling methods are Berendsen thermostat and Parrinello-Rahman, respectively. The Van der Waals (vdW) interactions were calculated by Cut-off method with the threshold of 1 nm. The electrostatic interactions were treated by Particle Mesh Ewald (PME) method. The final 5 ns was only sampled to make the analysis of radial distribution and coordinated structures. The diffusion coefficients of H atom in water were fitted by the slope of mean square displacement (MSD) and calculated by the Einstein equation, as listed in equation (8) and (9).

$$\mathrm{MSD} = \frac{[r(t-\Delta t)-r(t)]^2}{\Delta t} \quad (13)$$

$$D = \frac{1}{6} \lim_{t\to\infty} \left(\frac{d\mathrm{MSD}}{d\Delta t}\right) \quad (14)$$

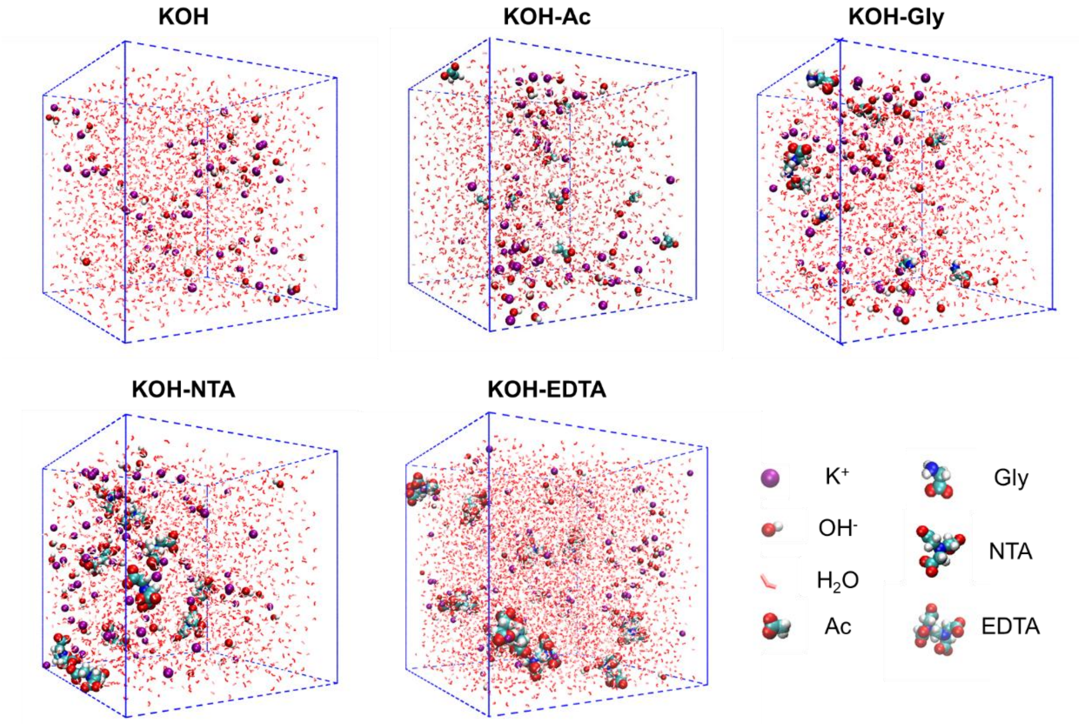

Figure S1. Snapshot of KOH solution with and without chelating agents.

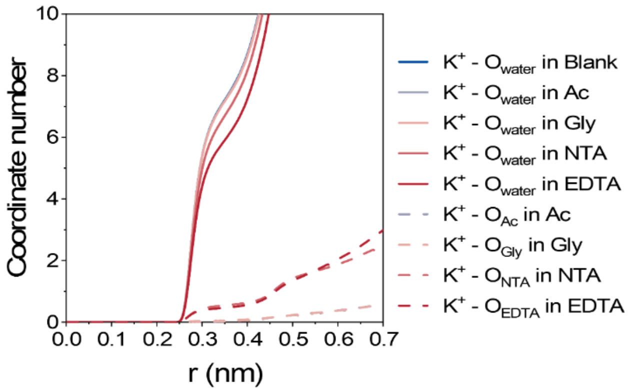

Figure S2. The coordination number of K^+-O_{EDTA} and K^+-O_{AC} collected from MD simulations. The specific values are listed in Supplementary Table 2.

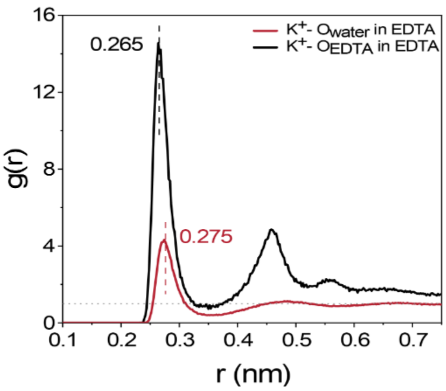

Figure S3. The radial distribution functions of K^+-O_{EDTA} and K^+-O_{water} in EDTA-containing electrolytes collected from MD simulations.

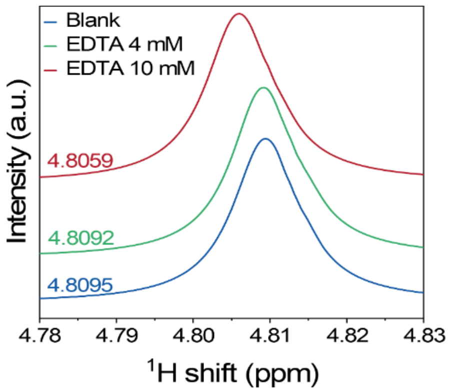

Figure S4. Experimental ^{1}H NMR spectra of 0.1 M KOH electrolytes with different EDTA content.

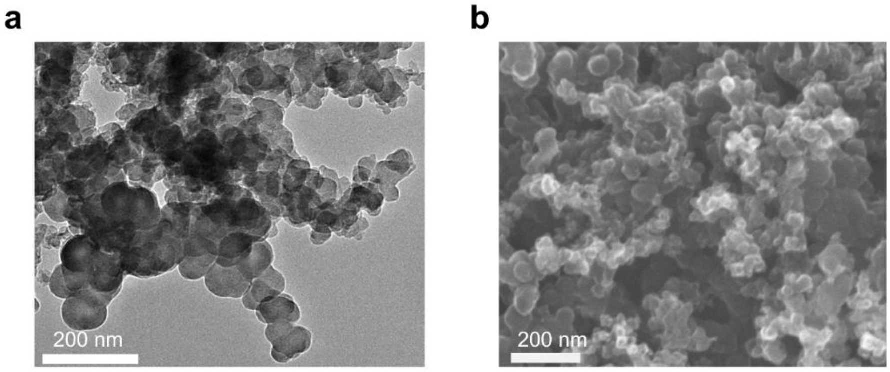

Figure S5. (a) TEM and (b) SEM image of carbon black (CB).

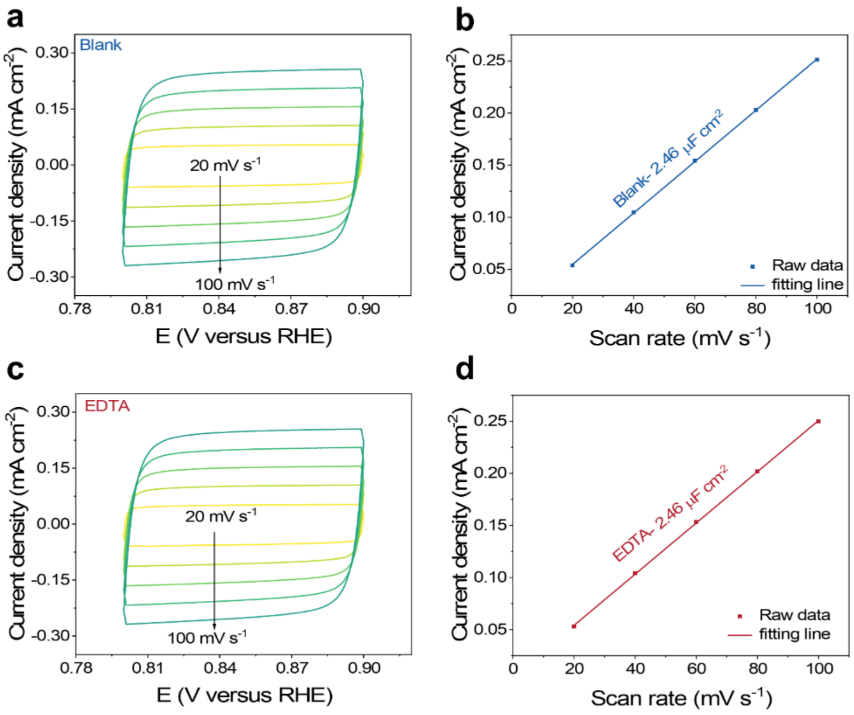

Figure S6. Electric double-layer capacitance (C_{dl}) measurement of CB in non-Faraday intervals (0.8 V_{RHE} - 0.9 V_{RHE}). (a) CV curves (scan rates: 20-100 mV s^{-1}) of CB in N_2-saturated 0.1 M KOH and (b) the C_{dl} calculation by plotting the current-density variation at 0.85 V_{RHE} against the scan rate to fit a linear regression. (c) CV curves (scan rates: 20-100 mV s^{-1}) of CB in N_2-saturated 0.1 M KOH electrolytes containing 4 mM EDTA and (d) the corresponding C_{dl} calculation.

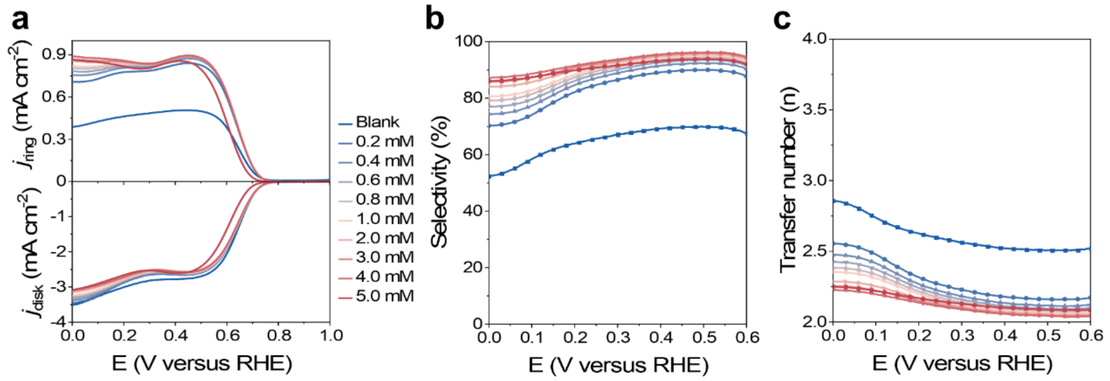

Figure S7. ORR activity and selectivity of CB catalysts in 0.1 M KOH electrolyte containing different concentrations of EDTA (0-5.0 mM). (a) LSV curves of CB catalysts at a scan rate of 5 mV s^{-1}. The ring current density j_{ring} represented the H_2O_2 oxidation current. (b) H_2O_2 selectivity and (c) the corresponding electron transfer number.

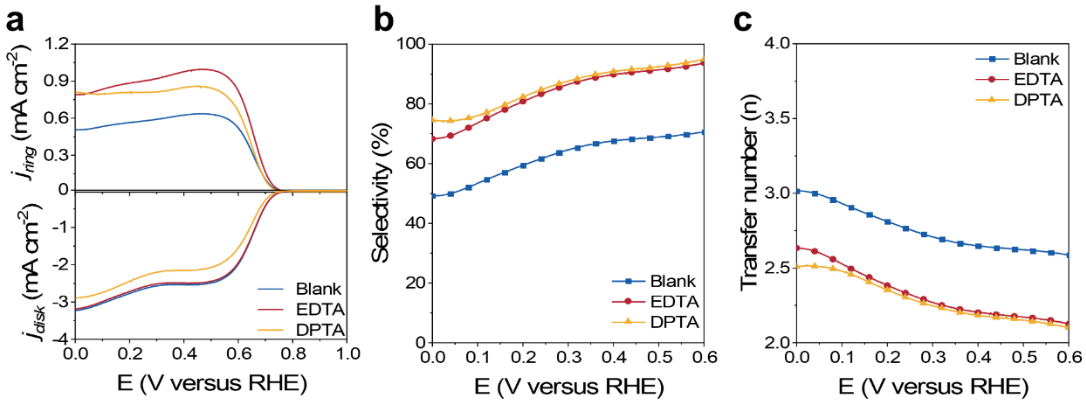

Figure S8. ORR activity and selectivity of CB catalysts in 0.1 M KOH electrolyte containing 4 mM DTPA or EDTA. (a) LSV curves of CB catalysts at a scan rate of 5 mV s^{-1}. The ring current density j_{ring} represented the H_2O_2 oxidation current. (b) H_2O_2 selectivity and (c) the corresponding electron transfer number of CB catalyst.

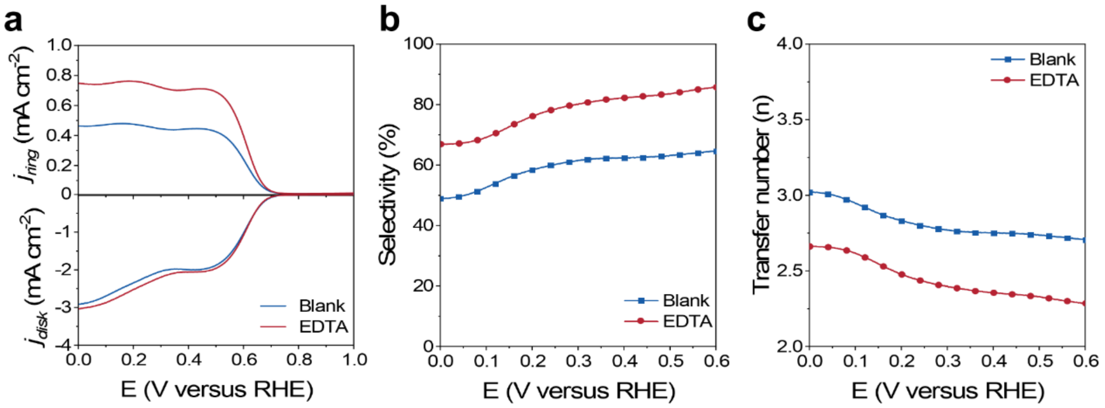

Figure S9. ORR activity and selectivity of CB in 0.1 M NaOH. (a) LSV curves of CB catalysts at a scan rate of 5 mV s^{-1} with or without 4 mM EDTA. The ring current density j_{ring} represented the H_2O_2 oxidation current. (b) H_2O_2 selectivity and (c) the corresponding electron transfer number of CB catalysts.

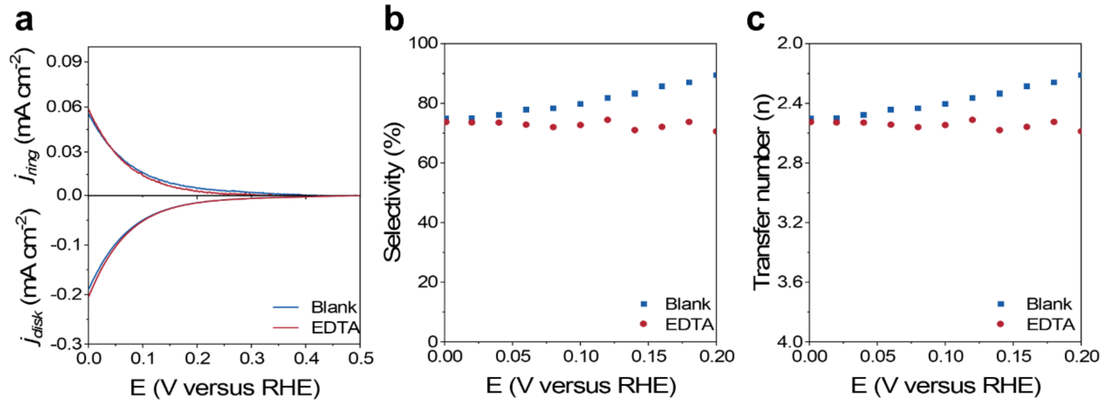

Figure S10. ORR activity and selectivity of CB catalysts in 0.1 M KOH electrolyte containing 4 mM DTPA or EDTA. (a) LSV curves of CB catalysts at a scan rate of 5 mV s^{-1}. The ring current density j_{ring} represented the H_2O_2 oxidation current. (b) H_2O_2 selectivity and (c) the corresponding electron transfer number.

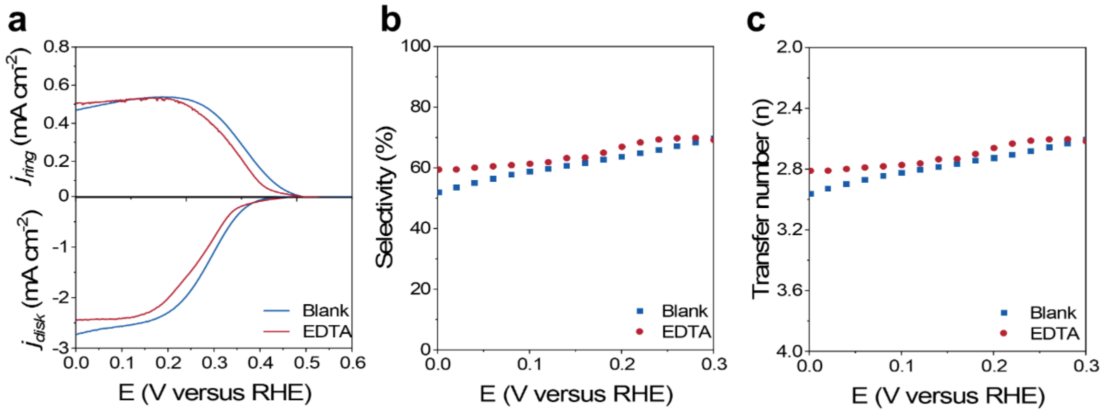

Figure S11. ORR activity and selectivity of CB in 0.05 M Na_2SO_4. (a) LSV curves of CB catalysts at a scan rate of 5 mV s^{-1} with or without 4 mM EDTA. The ring current density j_{ring} represented the H_2O_2 oxidation current. (b) H_2O_2 selectivity and (c) the corresponding electron transfer number.

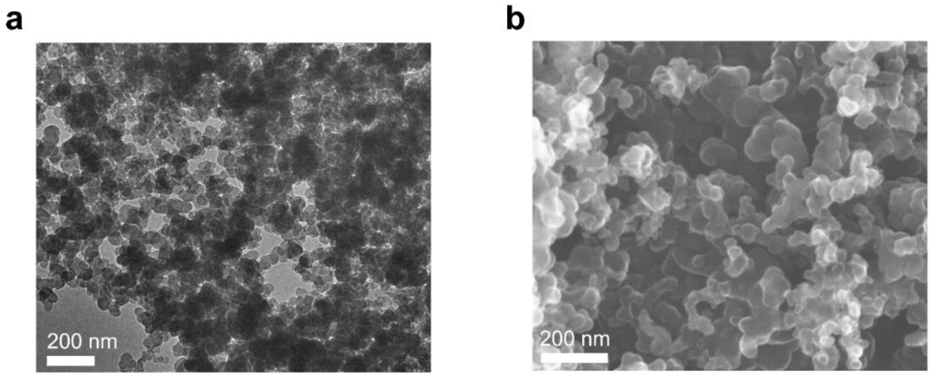

Figure S12. Characterization of Ketjen black. (a) TEM image. (b) SEM image.

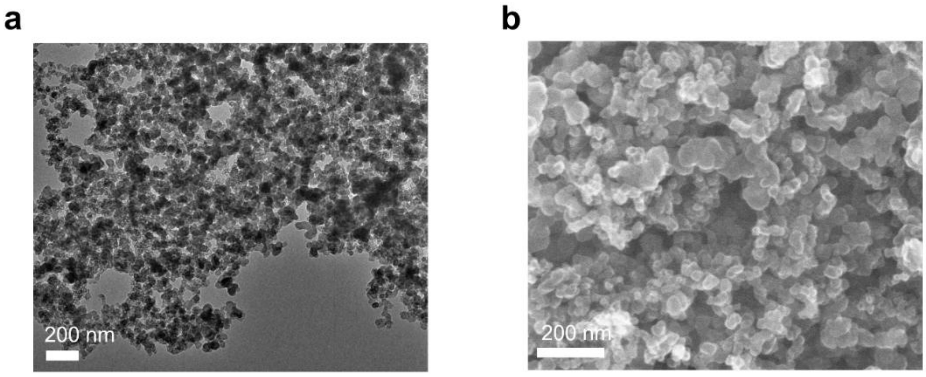

Figure S13. Characterization of acetylene black. (a) TEM image. (b) SEM image.

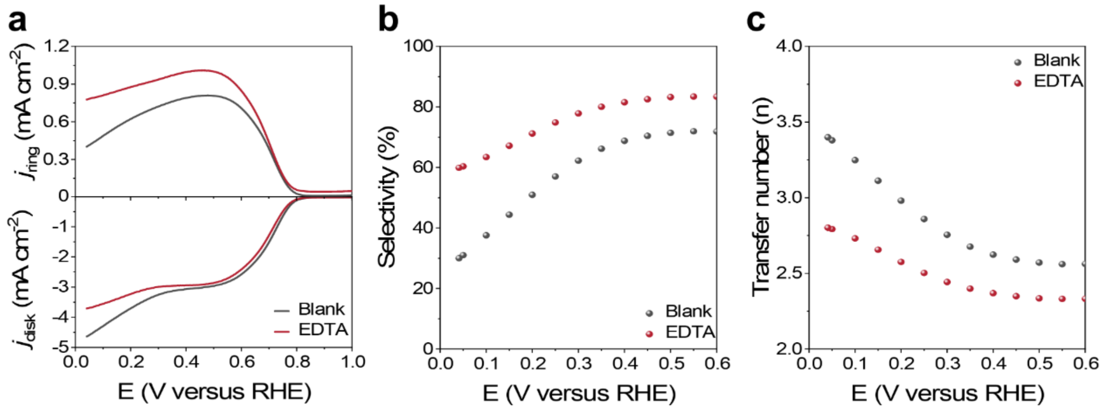

Figure S14. ORR activity and selectivity of Ketjen black. (a) LSV curves of Ketjen black catalysts at a scan rate of 5 mV s^{-1} in 0.1 M KOH and 0.1 M KOH electrolytes containing 4 mM EDTA. The ring current density j_{ring} represented the H_2O_2 oxidation current. (b) H_2O_2 selectivity and (c) the corresponding electron transfer number of Ketjen black catalysts.

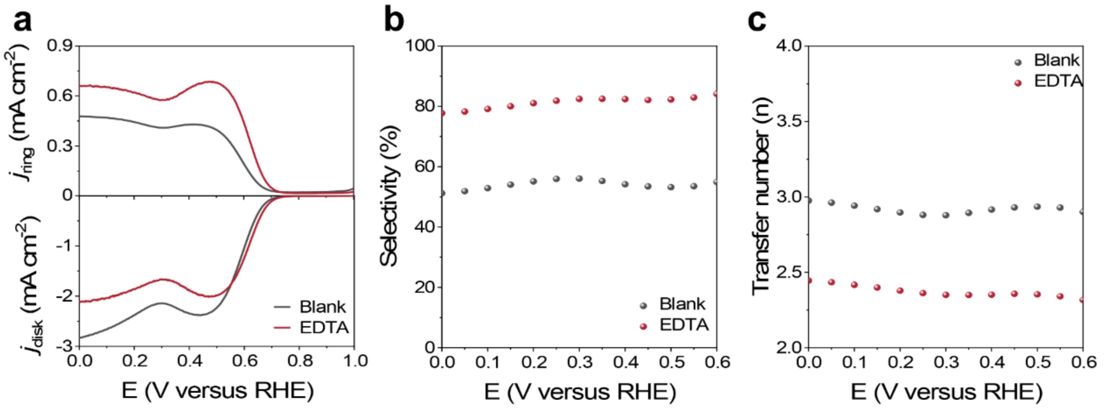

Figure S15. ORR activity and selectivity of acetylene black. (a) LSV curves of acetylene black catalysts at a scan rate of 5 mV s^{-1} in 0.1 M KOH and 0.1 M KOH electrolytes containing 4 mM EDTA. The ring current density j_{ring} represented the H_2O_2 oxidation current. (b) H_2O_2 selectivity and (c) the corresponding electron transfer number of acetylene black.

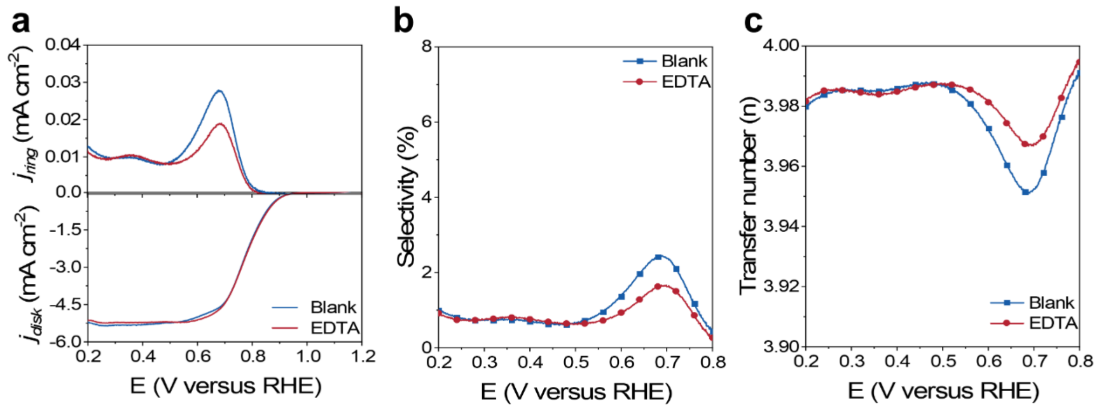

Figure S16. ORR activity and selectivity of Pt/C catalyst. (a) LSV curves of Pt/C at a scan rate of 5 mV s^{-1} in 0.1 M KOH and 0.1 M KOH electrolytes containing 4 mM EDTA. The ring current density j_{ring} represented the H_2O_2 oxidation current. (b) H_2O_2 selectivity and (c) the corresponding electron transfer number.

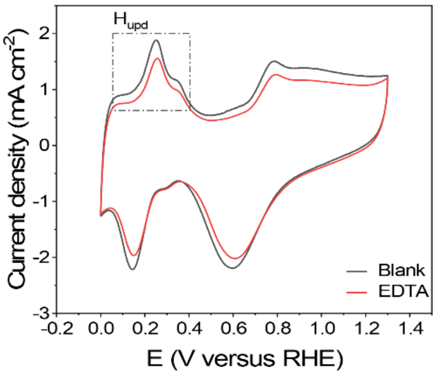

Figure S17. Cyclic voltammetry (CV) curves of the Pt/C catalyst in 0.1 M KOH solution before and after the addition of EDTA (4 mM) under a nitrogen atmosphere (scan rate: 50 mV s^{-1}, potential range: 0 – 1.2 V_{RHE}). The dashed region (0.05 – 0.40 V_{RHE}) represents the hydrogen underpotential adsorption (H_{upd}) interval, used to calculate the electrochemical surface area (ECSA) of Pt. The observed decrease in the integrated charge within the H_{upd} region after EDTA addition indicates strong coordination of EDTA to the Pt surface via its carboxyl groups, resulting in partial coverage of active sites.

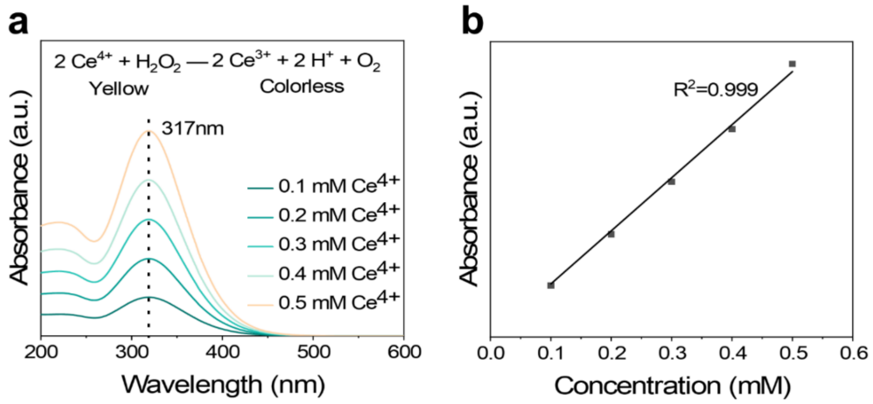

Figure S18. Standard curves of Ce^{4+} concentration. (a) UV-vis spectra of cerium solutions. (b) Corresponding fitted standard curve.

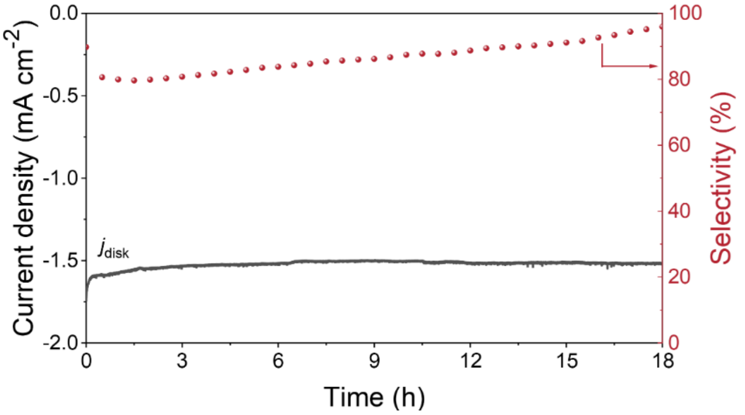

Figure S19. Stability test evaluated by RRDE in 0.1 M KOH electrolyte containing 4 mM EDTA under continuous oxygen bubbling. The disk electrode was held at 0.3 V_{RHE} while the Pt ring electrode was maintained at 1.2 V_{RHE} for real-time H_2O_2 detection.

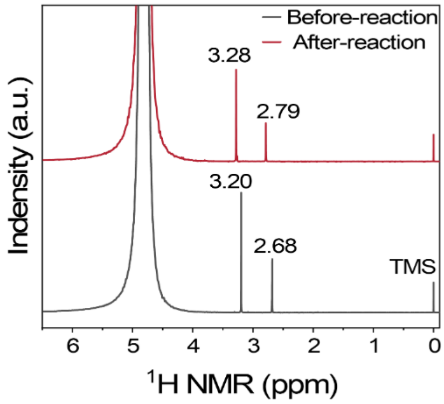

Figure S20. ^{1}H NMR spectra of the electrolyte before and after electrolysis. The electrochemical test was conducted at 0.3 V_{RHE} for 18 h in the presence of 4 mM EDTA. The characteristic peaks observed at δ 3.2 ppm and δ 2.7 ppm correspond to the ethylene (-CH=CH-) and methylene (-CH_2-) protons of EDTA, respectively. The minor shifts in these peaks after the reaction are attributed to the increased pH of the electrolyte due to electrolysis, rather than to structural degradation of EDTA[14].

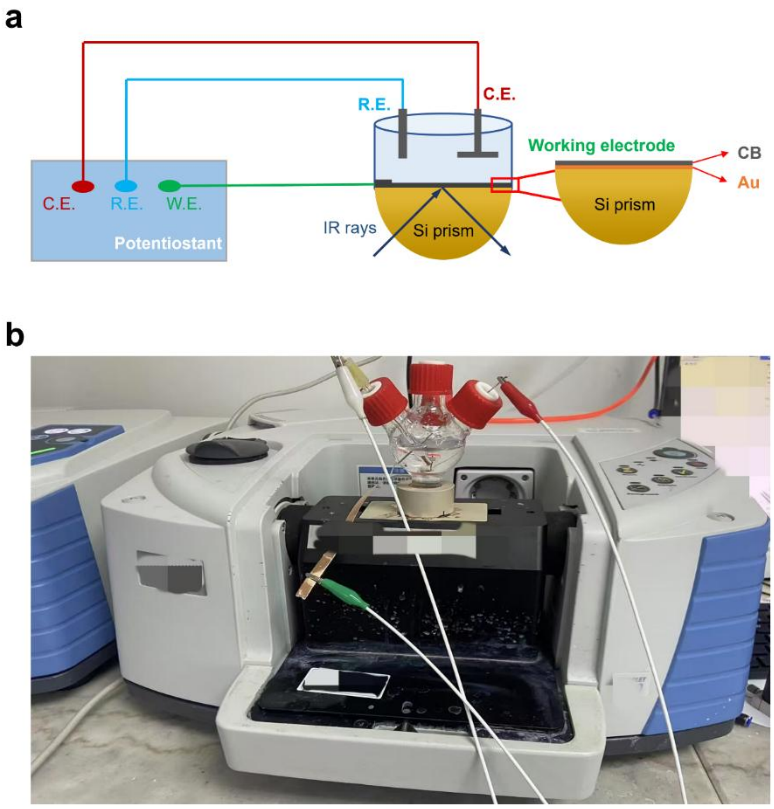

Figure S21. Photos of ATR-SEIRAS device. (a) Schematic diagram of ATR-SEIRAS device. (b) Photo of equipment taken during in-situ ATR-SEIRAS test.

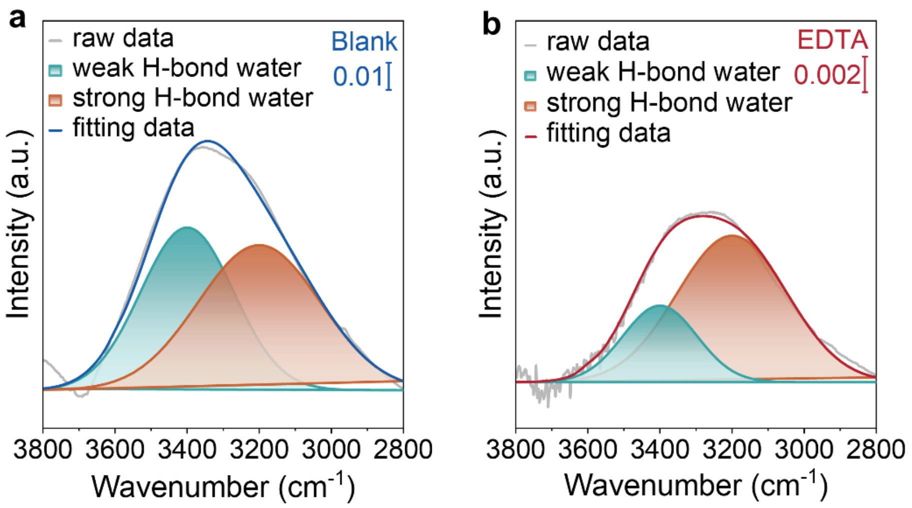

Figure S22. The fitting results of the ν-OH band in ATR-SEIRAS spectra of (a) the blank KOH electrolyte and (b) the KOH electrolyte containing 4 mM EDTA at 0 V_{RHE}. The detailed fitting data is shown in Table S3.

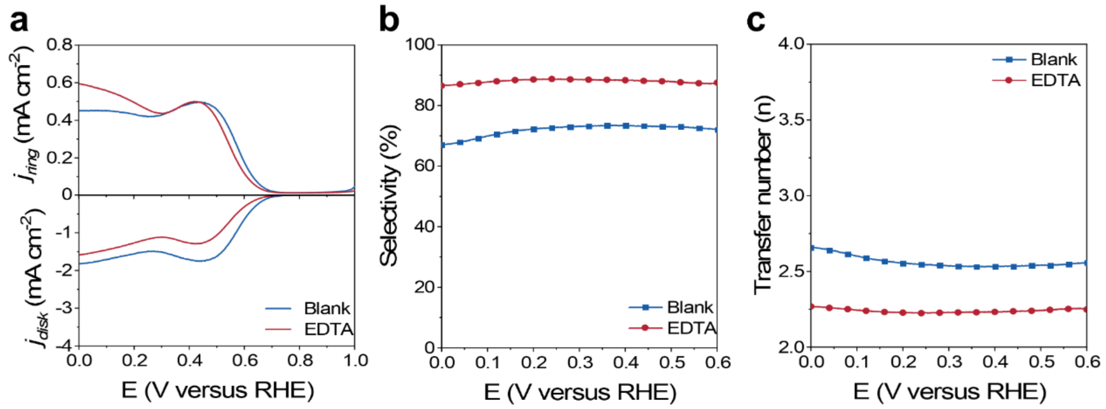

Figure S23. ORR activity and selectivity of glassy carbon electrode. (a) LSV curves of glassy carbon electrode at a scan rate of 5 mV s^{-1} in 0.1 M KOH and 0.1 M KOH electrolytes containing 4 mM EDTA. The ring current density j_{ring} represented the H_2O_2 oxidation current. (b) H_2O_2 selectivity and (c) the corresponding electron transfer number.

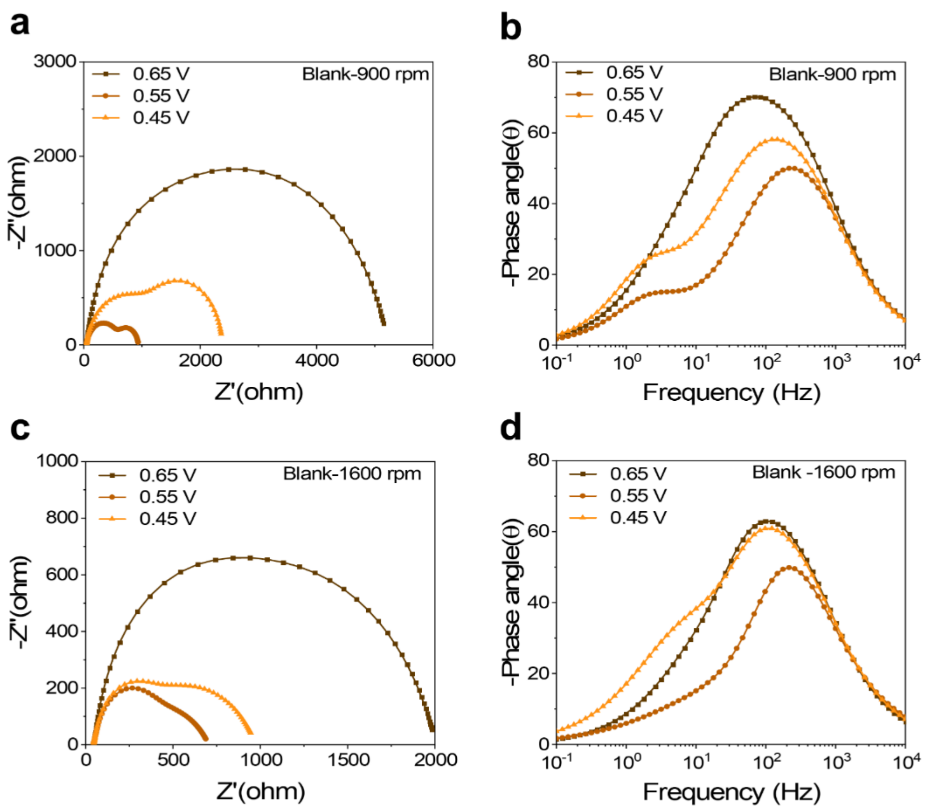

Figure S24. Electrochemical impedance spectra in 0.1 M KOH electrolyte. (a) Nyquist plots and (b) Bode plots at 0.65 – 0.45 V_{RHE} at a rotation rate of 900 rpm. (c) Nyquist plots and (d) Bode plots at 0.65 – 0.45 V_{RHE} at a rotation rate of 1600 rpm.

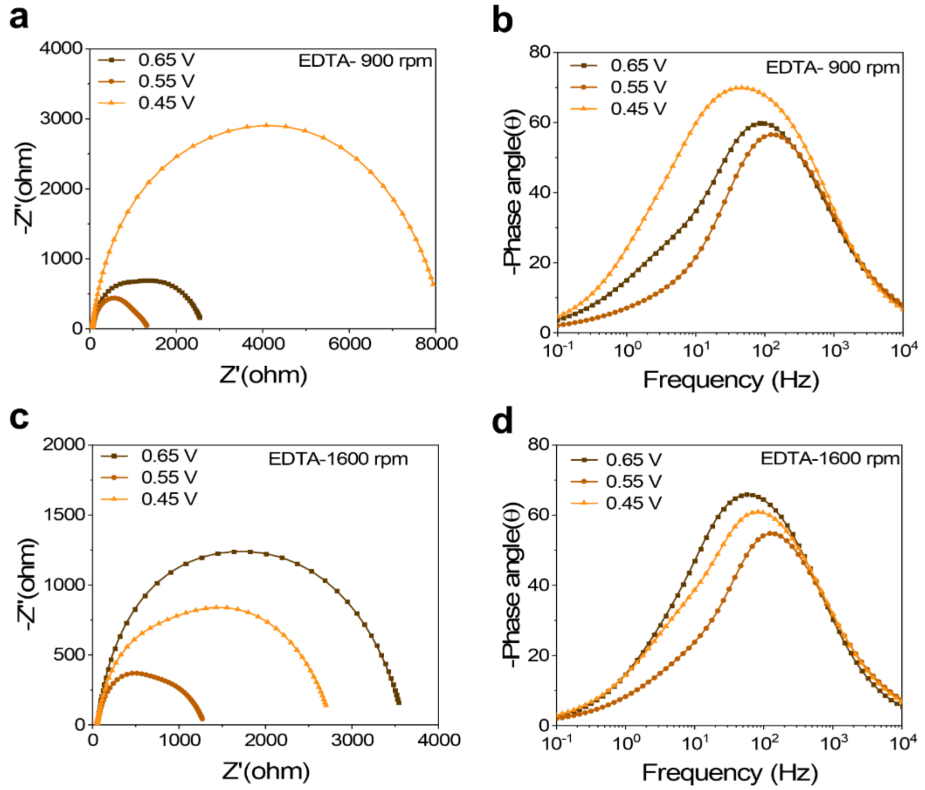

Figure S25. Electrochemical impedance spectra in 0.1 M KOH electrolytes containing 4 mM EDTA. (a) Nyquist plots and (b) Bode plots at 0.65 – 0.45 V_{RHE} at a rotation rate of 900 rpm. (c) Nyquist plots and (d) Bode plots at 0.65 – 0.45 V_{RHE} at a rotation rate of 1600 rpm.

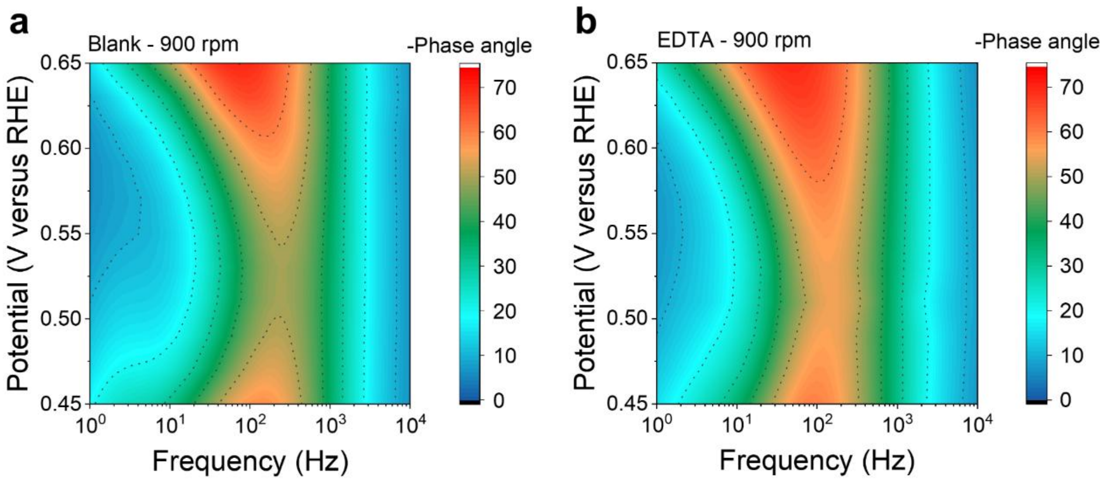

Figure S26. Bode plots with a working electrode rotation rate of 900 rpm. (a) Bode plots of in 0.1 M KOH electrolytes. (b) Bode plots of in 0.1 M KOH electrolytes containing 4 mM EDTA at 0.65– 0.45 V_{RHE}.

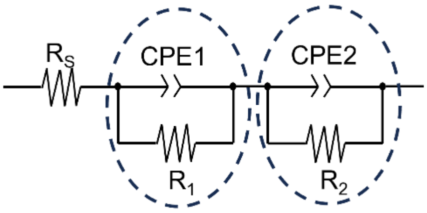

Figure S27. The equivalent circuit diagram. R_s is the solution resistance, CPE_1 and R_1 are related to the high-frequency charge transfer process, CPE_2 and R_2 are related to the high-frequency mass transfer process.

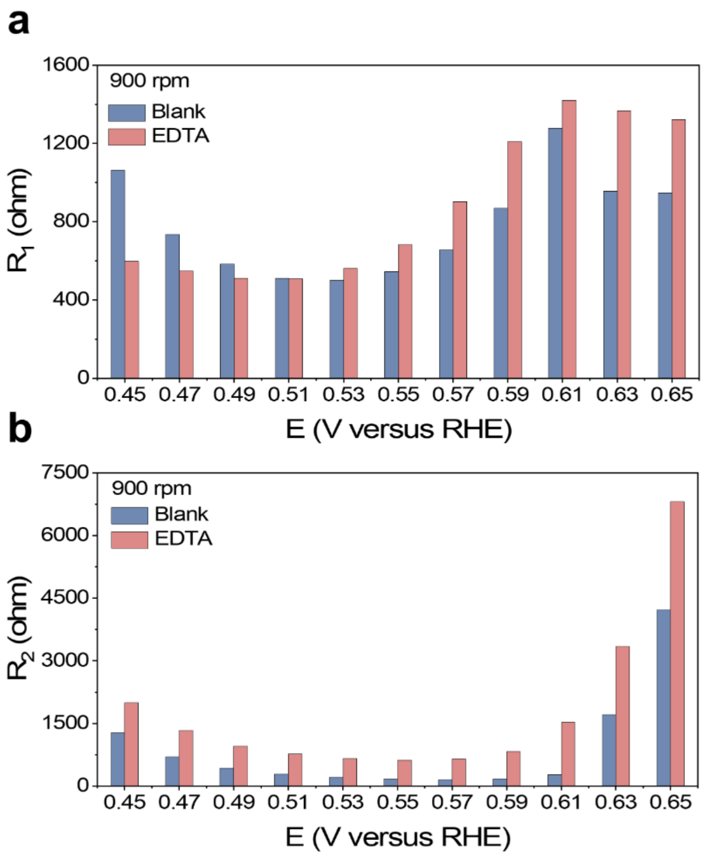

Figure S28. Fitting resistance from EIS measurements in 0.1 M KOH and 0.1 M KOH with 4 mM EDTA, recorded at a working electrode rotation rate of 900 rpm. (a) Charge transfer resistance R_1. (b) Mass transfer resistance R_2.

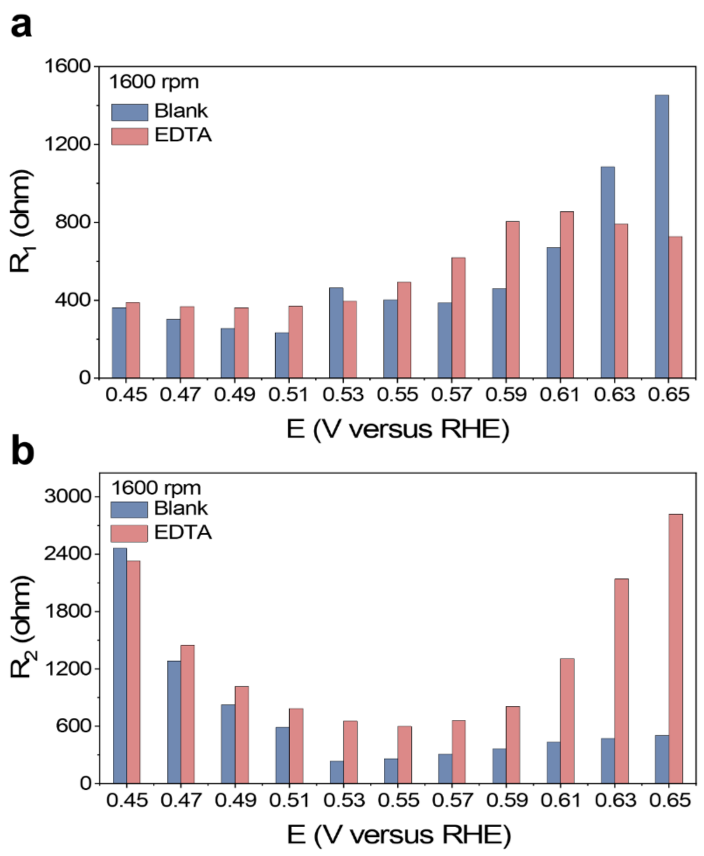

Figure S29. Fitting resistance from EIS measurements in 0.1 M KOH and 0.1 M KOH with 4 mM EDTA, recorded at a working electrode rotation rate of 1600 rpm. (a) Charge transfer resistance R_1. (b) Mass transfer resistance R_2.

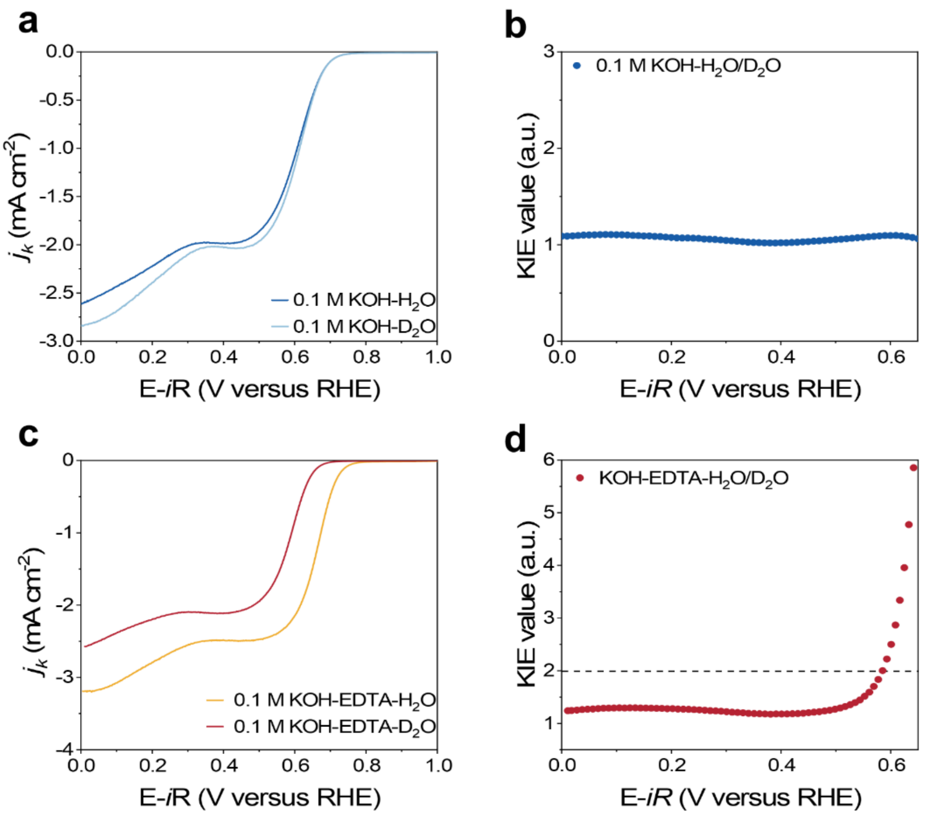

Figure S30. Kinetic isotope effect (KIE) measurements. (a) ORR polarization curves of CB obtained in 0.1 M KOH electrolyte prepared with H_2O and D_2O as solvents. (b) Corresponding KIE values for CB in 0.1 M KOH electrolyte. (c) ORR polarization curves of CB obtained in 0.1 M KOH electrolyte containing 4 mM EDTA prepared with H_2O and D_2O as solvents. (d) Corresponding KIE values for CB in 0.1 M KOH electrolyte containing 4 mM EDTA.

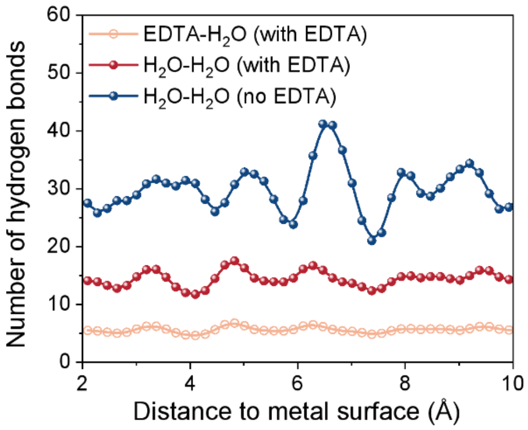

Figure S31. Statistical distribution of hydrogen bond number along the surface normal direction for KOH-H_2O and KOH-H_2O-EDTA systems.

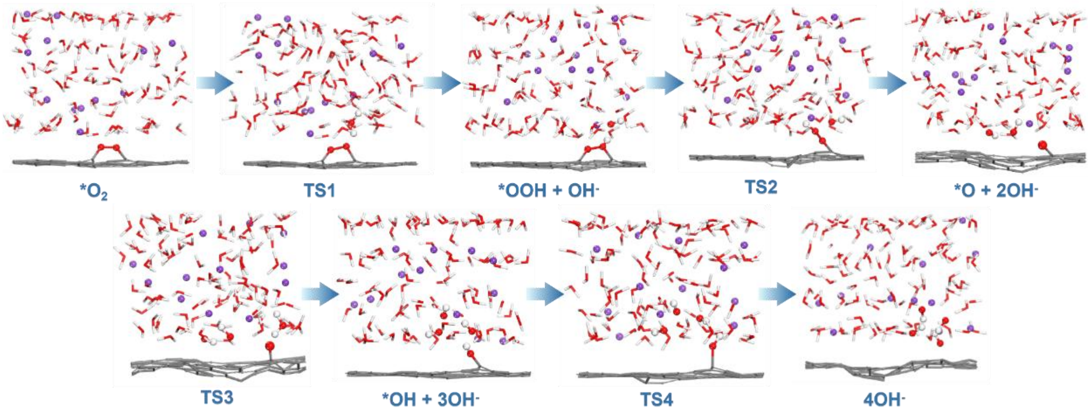

Figure S32. Representative snapshots of 4e^{-} ORR pathway at KOH-H_2O interfaces.

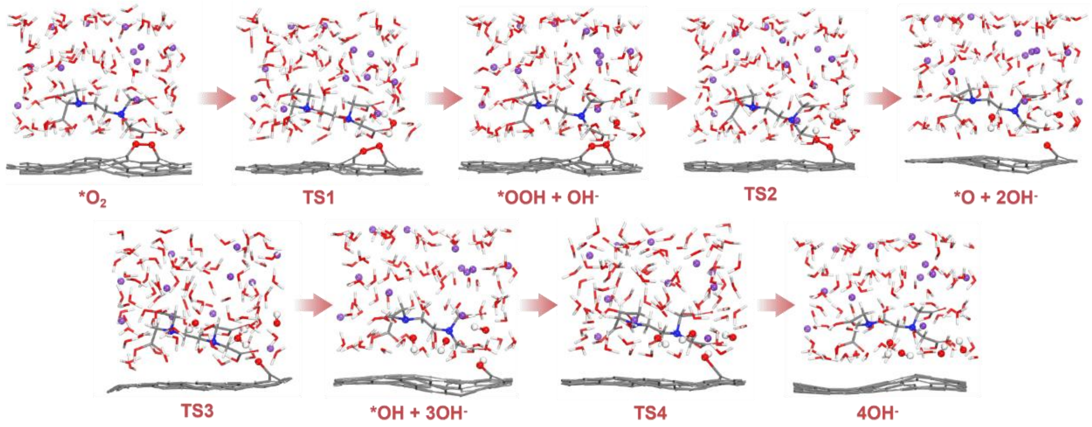

Figure S33. Representative snapshots of 4e⁻ ORR pathway at KOH-H_2O-EDTA interfaces.

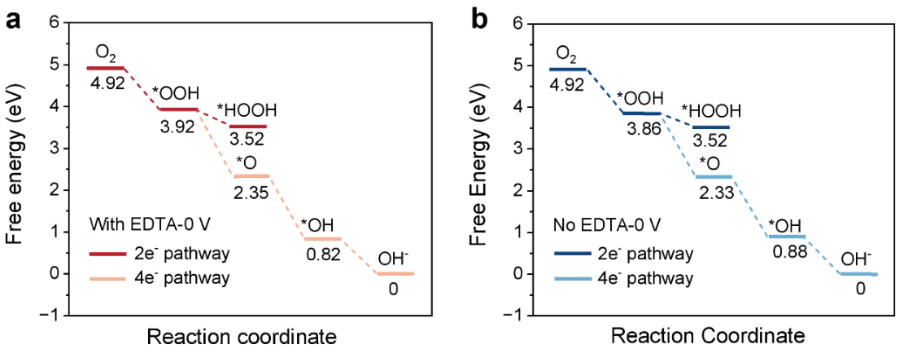

Figure S34. Free energy diagrams for 2e^{-} and 4e^{-} ORR at (a) KOH-H_2O-EDTA and (b) KOH-H_2O interfaces for U = 0 V vs RHE.

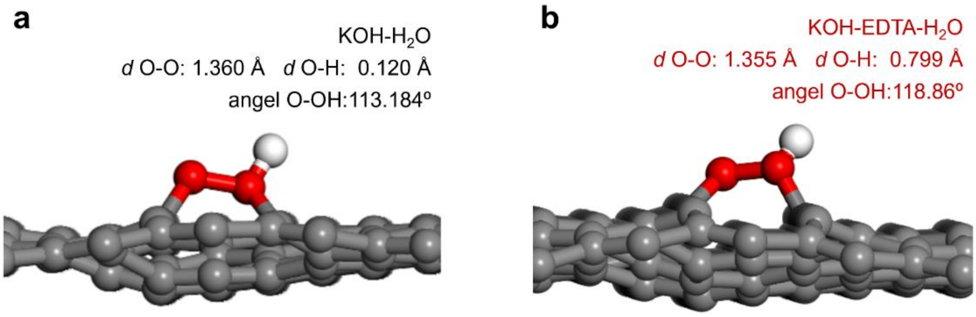

Figure S35. Comparison of adsorption configurations of *OOH, a key intermediate at TS2 in a stable state. The configurations depict *OOH in (a) KOH-H_2O and (b) KOH-EDTA-H_2O systems.

Table S1. Details of the simulated systems.

MD systems	K^+	OH^-	H_2O	targeted anions	Equilibrium volume (nm^3)	Equilibrium density ($kg\ m^{-3}$)
Blank	40	40	2113	-	62.49	1069.20
Ac	50	40	2070	10	62.56	1086.94
Gly	50	40	2064	10	62.00	1088.22
NTA	70	40	2012	10	61.23	1127.00
EDTA	80	40	1897	10	58.32	1157.00

In MD systems, the number of molecules or ions is shown in table above (K^+, OH^-, H_2O, targeted anions). The number of OH^- in each system did not change.

Table S2. Coordination number (CN) and coordination distance (*r*) in the different simulated systems.

MD systems	*r* (nm)	CN of K^+-O_{water}	CN of K^+-O_{CA}
Blank	0.358	7.395	-
Ac	0.358	7.372	0.050
Gly	0.358	7.345	0.061
NTA	0.360	6.931	0.556
EDTA	0.366	6.242	0.487

The radial distribution distance is defined as the minimum value corresponding to the distribution function (RDF). Then, based on the distribution distance, the specific combination number is obtained.

Table S3. Fitting parameters of diffusion coefficient in the simulated system.

MD systems	Slope of MSD ($Å^2$ ps^{-1})	R^2	D ($Å^2$ ps^{-1})
Blank	0.01769	0.99	0.02948
Ac	0.01665	0.99	0.02775
Gly	0.01624	0.99	0.02707
NTA	0.01517	0.99	0.02528
EDTA	0.01256	0.99	0.02093

The MSD slopes were obtained through linear fitting of the data in Fig. 2c, following Equations (12) and (13). The excellent fit quality ($R^2 > 0.99$ for all systems) confirms the validity of this approach. The resulting diffusion coefficients (Fig. 2d) and complete fitting parameters are summarized in the table above.

Table S4. Fitting parameters of interfacial water in 0.1 M KOH electrolyte.

Potential (V_{RHE})	A_1	A_2	a_1 (%)	a_2 (%)	R^2
0	20.48	19.68	51.00	49.00	0.99
0.1	18.78	18.63	50.20	49.80	0.99
0.2	11.12	5.48	66.97	33.03	0.99
0.3	3.32	8.49	71.86	28.14	0.99

A_1, A_2 correspond to the peak area of sub-peaks at 3200 cm^{-1}, 3400 cm^{-1}; a_1, a_2 are the area percentage of each sub-peak after normalizing the peak area; R^2 is the fitting coefficient.

Table S5. Fitting parameters of interfacial water in 0.1 M KOH electrolyte with 4 mM EDTA.

Potential (V_{RHE})	A_1	A_2	a_1 (%)	a_2 (%)	R^2
0	1.54	2.73	36.08	63.92	0.99
0.1	1.20	2.16	35.58	64.42	0.99
0.2	0.71	1.96	26.56	73.44	0.99
0.3	0.57	1.46	27.92	72.08	0.99

A_1, A_2 correspond to the peak area of sub-peaks at 3200 cm^{-1}, 3400 cm^{-1}; a_1, a_2 are the area percentage of each sub-peak after normalizing the peak area; R^2 is the fitting coefficient.

3. Supplementary References

1. Gu Y, Tan Y, Tan H *et al*. Industrial electrosynthesis of hydrogen peroxide over p-block metal single sites. *Nat Synth* 2025; **4**: 614-621.
2. Fan Y, Chen Y, Ge W *et al*. Mechanistic insights into surfactant-modulated electrode-electrolyte interface for steering H_2O_2 electrosynthesis. *J Am Chem Soc* 2024; **146**: 7575-7583.
3. Miyake H, Ye S, Osawa M. Osawa, M. Electroless deposition of gold thin films on silicon for surface-enhanced infrared spectroelectrochemistry. *Electrochem Commun* 2002; **4**: 973-977.
4. Tse ECM, Varnell JA, Hoang TTH *et al.* Elucidating proton involvement in the rate-determining step for Pt/Pd-based and non-precious-metal oxygen reduction reaction catalysts using the kinetic isotope effect. *J Phys Chem Lett* 2016; **7**: 3542-3547.
5. Kresse G, Hafner J. Ab initio molecular dynamics for liquid metals. *Phys Rev B* 1993; **47**: 558-561.
6. Blöchl, PE. Projector augmented-wave method. *Phys Rev B* 1994; **50**: 17953-17979.
7. Perdew JP, Burke K, Ernzerhof M. Generalized gradient approximation made simple. *Phys Rev Lett* 1996; **77**: 3865-3868.
8. Henkelman G, Jónsson H. Improved tangent estimate in the nudged elastic band method for finding minimum energy paths and saddle points. *J Chem Phys* 2000; **113**: 9978-9985.
9. Kresse G, Furthmüller J. Efficient iterative schemes for ab initio total-energy calculations using a plane-wave basis set. *Phys Rev B* 1996; **54**: 11169-11186.
10. Zhao Y, Schultz NE, Truhlar DG. Design of density functionals by combining the method of constraint satisfaction with parametrization for thermochemistry, thermochemical kinetics, and noncovalent interactions. *J Chem Theory Comput* 2006; **2**: 364-382.
11. Hess B, Kutzner C, Van Der Spoel D *et al.* GROMACS 4: Algorithms for highly efficient, load-balanced, and scalable molecular simulation. *J Chem Theory Comput* 2008; **4**: 435-447.
12. Becke AD. Density-functional thermochemistry: The effect of the exchange-only gradient correction. *J Chem Phys* 1992; **96**: 2155-2160.
13. Grimme S, Ehrlich S, Goerigk L. Effect of the damping function in dispersion corrected density functional theory. *J Comput Chem* 2011; **32**: 1456-1465.

14. Kula RJ, Sawyer DT, Chan SI *et al*. and Finley, C M. Nuclear magnetic resonance studies of metal-ethylenediaminetetraacetic acid complexes. *J Am Chem Soc* 1963; **85**: 2930-2936.
